# New lens on congenital mild bone fragility: a novel *Col1a1* knockout mouse model for osteogenesis imperfecta type 1

**DOI:** 10.1093/jbmr/zjaf138

**Published:** 2025-10-06

**Authors:** Lidiia Zhytnik, Laura Ventura, Anastasia Sclocco, Matthjis Verhage, Astrid D Bakker, Jae-Hyuck Shim, Wissam Beaino, Pedro M Pereira, Myrthe E Hoogeland, Vivi M Heine, Huub Maas, Richard T Jaspers, Anja Niehoff, Frank Zaucke, Vivian de Waard, E Marelise W Eekhoff, Dimitra Micha

**Affiliations:** Department of Traumatology and Orthopaedics, The University of Tartu, 50410 Tartu, Estonia; Department of Human Genetics, Amsterdam UMC Location VUmc, 1081 HZ Amsterdam, The Netherlands; Rare Bone Disease Center Amsterdam, 1081 HV Amsterdam, The Netherlands; Amsterdam Reproduction and Development, Amsterdam, The Netherlands; Amsterdam Movement Sciences, Amsterdam, The Netherlands; Department of Human Genetics, Amsterdam UMC Location VUmc, 1081 HZ Amsterdam, The Netherlands; Rare Bone Disease Center Amsterdam, 1081 HV Amsterdam, The Netherlands; Amsterdam Reproduction and Development, Amsterdam, The Netherlands; Amsterdam Movement Sciences, Amsterdam, The Netherlands; Department of Human Genetics, Amsterdam UMC Location VUmc, 1081 HZ Amsterdam, The Netherlands; Department of Human Genetics, Center for Neurogenomics and Cognitive Research, University Medical Center Amsterdam, 1081 HV Amsterdam, The Netherlands; Department of Oral Cell Biology, Academic Centre for Dentistry Amsterdam (ACTA), University of Amsterdam and Vrije Universiteit Amsterdam, 1081 LA Amsterdam, The Netherlands; Department of Cellular and Genetic Medicine, Horae Gene Therapy Center, University of Massachusetts Medical School, Worcester, MA 01655, United States; Department of Radiology and Nuclear Medicine, UMC Location, Vrije Universiteit Amsterdam, 1081 HV, Amsterdam, The Netherlands; Amsterdam Neuroscience, Brain Imaging, Amsterdam, The Netherlands; Department of Radiology and Nuclear Medicine, UMC Location, Vrije Universiteit Amsterdam, 1081 HV, Amsterdam, The Netherlands; Amsterdam Neuroscience, Brain Imaging, Amsterdam, The Netherlands; Department of Medical Biochemistry, Amsterdam UMC Location, University of Amsterdam, Amsterdam Cardiovascular Sciences, Atherosclerosis and Ischaemic Syndromes, 1105 AZ Amsterdam, The Netherlands; Department of Child and Adolescent Psychiatry, Amsterdam UMC Location, Vrije Universiteit Amsterdam, Amsterdam Neuroscience, 1081 HV Amsterdam, The Netherlands; Department of Complex Trait Genetics, Center for Neurogenomics and Cognitive Research, Vrije Universiteit Amsterdam, Amsterdam Neuroscience, 1081 HV Amsterdam, The Netherland; Amsterdam Movement Sciences, Amsterdam, The Netherlands; Department of Human Movement Science, Faculty of Behavioral and Movement Sciences, Vrije Universiteit Amsterdam, 1081 HZ Amsterdam, The Netherlands; Amsterdam Movement Sciences, Amsterdam, The Netherlands; Department of Human Movement Science, Faculty of Behavioral and Movement Sciences, Vrije Universiteit Amsterdam, 1081 HZ Amsterdam, The Netherlands; Institute of Biomechanics and Orthopaedics, German Sport University Cologne, 50933 Köln, Germany; Cologne Center for Musculoskeletal Biomechanics, Faculty of Medicine and University Hospital Cologne, University of Cologne, 50931 Köln, Germany; Dr. Rolf M. Schwiete Research Unit for Osteoarthritis, Department of Trauma Surgery and Orthopaedics, University Hospital, Goethe University Frankfurt, 60528 Frankfurt am Main, Germany; Department of Medical Biochemistry, Amsterdam UMC Location, University of Amsterdam, Amsterdam Cardiovascular Sciences, Atherosclerosis and Ischaemic Syndromes, 1105 AZ Amsterdam, The Netherlands; Rare Bone Disease Center Amsterdam, 1081 HV Amsterdam, The Netherlands; Amsterdam Reproduction and Development, Amsterdam, The Netherlands; Amsterdam Movement Sciences, Amsterdam, The Netherlands; Department of Endocrinology and Metabolism, Amsterdam UMC Location Vrije Universiteit, 1081 HV Amsterdam, The Netherlands; Department of Human Genetics, Amsterdam UMC Location VUmc, 1081 HZ Amsterdam, The Netherlands; Rare Bone Disease Center Amsterdam, 1081 HV Amsterdam, The Netherlands; Amsterdam Reproduction and Development, Amsterdam, The Netherlands; Amsterdam Movement Sciences, Amsterdam, The Netherlands

**Keywords:** *Col1a1*, bone, collagen, haploinsufficiency, transgenic mouse model, osteogenesis imperfecta, knockout

## Abstract

Osteogenesis imperfecta (OI) is a genetic disorder characterized by bone fragility. It is one of the most prevalent rare skeletal dysplasias. The mildest form, OI type 1, predominantly results from collagen type I haploinsufficiency due to pathogenic variants in the *COL1A1* gene, leading to reduced collagen type I. Despite OI type 1 representing approximately half of the OI population, the lack of an effective mouse model has hindered research and therapy development. To address this gap, we developed a genetically engineered mouse model harboring a heterozygous deletion of the *Col1a1* allele using the CRISPR/Cas system. The bone phenotype was characterized in 8- and 24-wk-old mice, assessing transcriptomics and serum markers for bone formation (procollagen type I N-terminal propeptide) and resorption (tartrate-resistant acid phosphatase 5b). Bone volume, microarchitecture, and strength were evaluated by micro-CT, histomorphometry, and three-point bending test. We showed that the decreased *Col1a1* to *Col1a2* mRNA ratio determines reduced collagen type I production in OI mice bones as the underlying mechanism of haploinsufficient OI. This was supported by *COL1A1* to *COL1A2* mRNA ratio findings in human OI cell models, including fibroblasts and induced mesenchymal stem cells, as well as in induced pluripotent and mesenchymal stem cell models that were edited to carry a heterozygous *COL1A1* allele. Our findings indicate for the first time that reduced bone volume and altered bone microarchitecture in haploinsufficient OI depends on the *Col1a1* to *Col1a2* mRNA ratio regulation. This novel mouse model faithfully recapitulates OI type 1 and provides a vital tool for investigating the disease mechanism and developing targeted therapeutic strategies for this large neglected OI patient population.

## Introduction

Osteogenesis imperfecta (OI) is a rare genetic disorder characterized by congenital bone fragility, with an incidence of 1 in 10 000 to 15 000 births. Along with achondroplasia, OI is one of the most prevalent forms of rare skeletal dysplasia.^1^ Approximately 85% of OI patients harbor pathogenic variants in the *COL1A1* (OMIM 120150) or *COL1A2* (OMIM 120150) genes.^2^ These genes encode the pro-α1(I) and pro-α2(I) chains, respectively, which combine in a 2:1 ratio to form a heterotrimer by intertwining into triple-helix molecules that assemble into collagen type I fibrils.^3^ This structure provides tensile strength and flexibility, enabling bones to withstand mechanical stress and strain. Collagen type I is a critical component of the bone matrix, essential for maintaining the structural integrity and strength of bones.^4^ As the main organic component of bone, it serves as a scaffold for the deposition of hydroxyapatite crystals, crucial for bone mineralization.^5^ Additionally, collagen type I regulates bone formation, maintenance, and remodeling through its interactions with bone cells and other bone matrix proteins, making it indispensable for the functional performance of bone tissue.^6^

Osteogenesis imperfecta is clinically variable, ranging from mild osteopenia to progressive skeletal deformities. The clinical Sillence classification of OI identifies five main types: type 1 (OMIM 166200, 166240)—non-deforming, mild bone fragility with blue sclerae; type 2 (OMIM 166210)—perinatally lethal; type 3 (OMIM 259420)—progressively deforming with severe skeletal fragility; type 4 (OMIM 166220)—moderate bone fragility with white sclerae; and type 5 (OMIM 610967)—moderate bone fragility with ectopic ossification.^7^^,^^8^ Osteogenesis imperfecta type 1 is the most prevalent form, caused by collagen type I haploinsufficiency (HI) due to pathogenic variants in the *COL1A1* gene, and to a lesser extent by less deleterious structural variants.^2^^,^^9–12^ Haploinsufficient OI accounts for a striking 52% of the OI patient population and arises from heterozygous whole gene deletions, nonsense mutations, out-of-frame frameshifts, and certain splice site variants.^2^^,^^9–13^ This results in quantitative deficiency of collagen type I, which leads to bone fragility but less severe skeletal deformities and clinical course compared to qualitative collagen defect-related OI.^10^^,^^14^^,^^15^ Despite the relatively high prevalence of this rare disorder, research and therapeutic advances for HI OI type 1 have been significantly hindered by the lack of an adequate mouse model.^16^ Most mouse models focus on severe forms of OI, and some recapitulate *Col1a1*-related OI with varying degrees of severity: G859C (OI type 2), Aga2 (OI type 2, 3), Brtl (OI type 2, 4), human COL1A1-minigene (OI type 2-4), Jrt (OI type 4, EDS), Mov13 (OI type 1),^16^^,^^17^ Col1a1^+/−^ (OI type 1),^18^ and Col1a1^±365^ (OI type 1).^17^^,^^19^ The latter three mimic HI OI: the Col1a1^±365^ (365 bp deletion) and Col1a1^+/−^ mice carry a heterozygous knockout of exon 2 to exon 5, resulting in a premature termination codon and collagen type I HI; the Mov13 mouse carries an insertion of a Moloney murine leukemia virus genome in intron 1, interfering with *Col1a1* transcription.^16^^,^^18^^,^^19^ Due to alterations in the regulatory intronic region, Mov13 mice exhibit tissue-specific inconsistency in collagen type I expression and develop severe leukemia at early age, making them unsuitable for elucidating the pathological mechanisms and therapy testing for HI OI.^16^ The Col1a1^±365^ and Col1a1^+/−^ mice,^18^^,^^19^ in turn, have a premature stop codon, which is known to be biologically suppressed in a small percentage of cases, suggesting a possible effect of truncated protein on bone pathophysiology.^20^ Consequently, there is no existing true heterozygous *Col1a1* knockout mouse model that accurately reflects collagen type I HI. Therefore, this study aims to develop and characterize a novel genetically engineered mouse model with collagen type I HI, named the hiOI mouse model. This model exhibits a fragile bone phenotype, thereby addressing the current gap in available models for this common form of OI. This model will serve as a valuable tool for elucidating the pathological mechanisms and advancing research and therapeutic strategies for HI OI.

## Materials and methods

### Study design

All animal experiments were reviewed and approved by the Central Committee of Animal experiments (CCD) of the Netherlands. Ad libitum feeding and group housing were employed. Procedures were performed in accordance with the guidelines of the Animal Welfare Body of Amsterdam UMC and in full compliance with the directive 2010/63/EU. For the experiments, each age group (8 and 24 wk) included 20 hiOI and 20 WT mice (50% male), unless otherwise stated. Details on the age, sex, and number of mice used in each experiment are provided in Table S1.

### Generation of a *Col1a1* knockout mouse model

**Figure 1:**
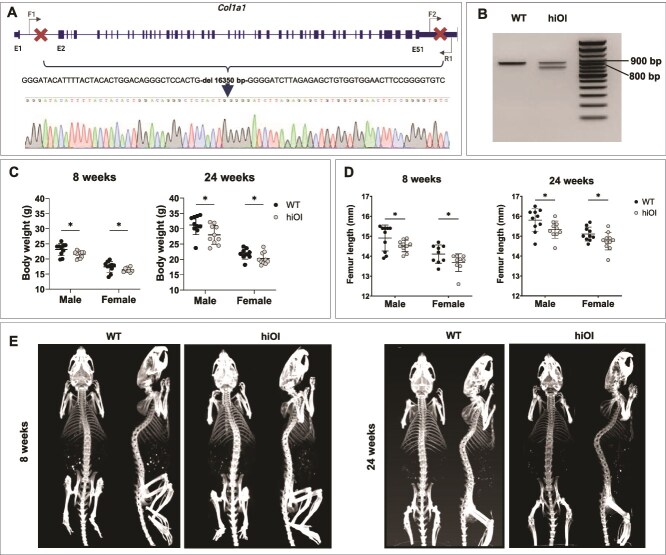
Generation of a novel *Col1a1* knockout mouse model for haploinsufficient OI (hiOI). (A) Knockout strategy with a 16 350 bp deletion in the *Col1a1* gene for the hiOI mouse and Sanger sequencing of the mutated allele. Created with Biorender.com. (B) PCR products of WT mouse—only the 890 bp WT allele present—and hiOI mouse—two alleles of 765 bp and 890 bp are produced. (C) Reduced body weight in 8- and 24-wk-old male and female hiOI mice and their WT littermates (^*^p ≤ .05; data shown as mean ± SD). (D) Femora of hiOI mice were shorter compared to WT controls at the age of 8 and 24 wk (^*^p ≤ .05; data shown as mean ± SD). (E) CT images of the whole skeleton of WT and hiOI mice showing absence of visible skeletal dysplasia or fractures. Images of male WT and hiOI mice at the age of 8 wk.

Mice were created by Cyagen Biosciences. The gRNA sequences targeting the mouse *Col1a1* gene were: TGTGGAGGGGCTGATTTCAGTGG (forward strand) and CCTCAAGGAACTCTCCCCGGGGG (reverse strand). Off-target sites were predicted using CCTop (https://cctop.cos.uni-heidelberg.de/), and validation was performed at the DNA level for the top 5 sites to check for cleavage. In the absence of cleavage, off-target effects were excluded. The gRNA efficacy was also predicted using CCTOp based on the CRISPRater scores (low: score < 0.56; medium: 0.56 ≤ score ≤ 0.74; high: score > 0.74) and confirmed by results from F0 positive individuals. The gRNAs and Cas9 mRNA were co-injected into C57BL/6N mouse zygotes to generate targeted knockout offspring. Injected zygotes were implanted in pseudo-pregnant females. Positive founders were bred to produce F1 generation, which was subsequently crossed with WT mice. Animals were genotyped by PCR followed by Sanger sequencing analysis, using the following primers: forward primer 1 (F1): 5′-AGAAAGTTTGGCTAGGACTTGACT-3′, forward primer 2 (F2): 5′-CTCACTTCTCATCCAGATATTGCCA-3, and reverse primer (R1): 5′-TTCTACTATGTCACCGTCCCCATT-3′ (Figure 1A, Supplementary material).

### Tissue preparation

Mice were weighed and terminated at 8 or 24 wk of age by CO_2_-induced hypoxia. Organs of interest (blood serum, humeri, femora, and tibiae) were collected. Blood was drawn via retro-orbital collection and centrifuged twice at 4 °C for 10 min at 1000 × *g*, and serum was stored at −20 °C. Bones were cleaned of soft tissue. The heads of the humeri and the right femur were cleaved off, and bone marrow was removed by centrifugation and PBS washes. Humeri and right femora were stored in RNAlater-ICE solution (Thermo Fisher Scientific) at −20 °C before RNA isolation. Left femora were stored at −20 °C in PBS-soaked gauze prior to micro-CT analysis and three-point bending test. Left tibiae were fixed in 4% paraformaldehyde for 24 h and stored in 70% ethanol at 4 °C.

### Computed tomography

Full-body CT scans of hiOI and WT male and female mice were performed post-mortem in prone position. Scans were conducted using a Mediso nanoScan system (Mediso Ltd.). The acquisition was performed with 480 projections and a semi-circular scanning method. The parameters included 50 kVp X-ray energy, a 300 ms exposure time, and 1:4 binning. CT images were reconstructed by the Nucline software (Mediso Ltd.) using the Filtered Back Projection (FBP) algorithm.

### Micro-CT

Full-length left femora were scanned with the SkyScan 1272 CMOS (Bruker, version 1.4) using the following settings: 12 μm resolution, 2016 × 1344 pixels, filter: Al 0.5 mm. Each sample was rotated 360° in increments of 0.2°. The images were reconstructed using the NRecon software (Bruker, version 1.7.4.2). The trabecular volume of interest (VOI) was located in the distal femur containing secondary spongiosa (thickness of ~2.4 mm). For the cortical VOI, a section of 2.0 mm of the midshaft was analyzed. CTan (Bruker, version 1.20.8.0) software was used for the examination and quantification of BMD and bone three-dimensional (3D) parameters. CT.vox (Bruker) was used for visualizing the reconstructed bone images. Trabecular bone parameters, including trabecular bone volume (Tb.BV) and volume fraction (Tb.BV/TV), bone surface density (Tb.BS/TV), thickness (Tb.Th), number (Tb.N), separation (Tb.S), width (Tb.Wi), and connectivity density (Conn.Dn) were assessed. In the cortical bone, the parameters measured included cortical bone volume fraction (Ct.BV/TV), thickness (Ct.Th), width (Ct.Wi), total cross-sectional area inside the periosteal envelope (Tt.Ar), bone area (Ct.Ar), and area fraction (Ct.Ar/Tt.Ar) and bone perimeter to tissue area (Ct.B.Pm/T.Ar). All measured parameters were body weight-corrected using the linear regression method described by Jepsen et al.^21^

### Three-point bending test

In order to analyze the mechanical properties of the left femora, three point bending tests were performed using a materials testing machine (Z2.5/TN1S; ZwickRoell GmbH & Co. KG), as described previously.^22^ In brief, femora were loaded at mid-diaphysis in anterior–posterior direction using a 100-N load cell. The distance between support points was 5 mm. The loading surfaces had a curvature with a radius of 0.75 mm. After pre-loading at 0.1 N and a loading rate of 0.05 mm/s the bones were loaded to failure with a crosshead speed of 1 mm/min. Ultimate load (N), deformation (mm), stiffness (N/mm), and energy (mJ) were measured from the load-deformation curve. Ultimate stress (MPa) and ultimate strain (%), elastic modulus (MPa), as well as energy density (mJ/mm^3^) were calculated based on the area moments of inertia obtained from micro-CT measurements.

### Histomorphometry

The tibiae of 8-wk-old male hiOI and WT mice were processed through a series of alcohol dehydrations and subsequently infiltrated with methyl methacrylate (Merck, 800590) supplemented with 99% dibutyl phthalate (Merck, 524980) and Perkadox 16 (Nouryon, 15520-11-3). The tibiae were positioned horizontally at the bottom of glass jars for the embedding process. Longitudinal sections of 5 μm thickness were cut with a Jung model “K” microtome (Jung) at three different levels, with 20 μm intervals between each level, and mounted onto gelatin-coated glass slides (VWR). Methyl methacrylate-embedded sections were stained for Masson-Goldner trichrome staining. Bright-field images were captured using a VS200 Olympus Slide Scanner (Olympus Life Science) with the 20x objective magnification and analyzed using QuPath software v0.5.1.

The primary histomorphometric parameters measured included cortical width (Ct.Wi), tissue area (T.Ar), bone area (B.Ar), bone perimeter (B.Pm), osteocyte number (N.Ot/B.Pm), and osteoid perimeter (O.Pm/B.Pm). Cortical width (Ct.Wi) was measured at least two times at each site of the bone shaft. The trabecular bone was evaluated in a defined area 200 μm below the growth plate, and half of Ct.Wi from the cortical bone, in order to exclude primary spongiosa and endocortical bone. The trabecular bone parameters measured include trabecular bone volume fraction (Tb.BV/TV), trabecular bone surface density (Tb.BS/TV), trabecular thickness (Tb.Th), trabecular number (Tb.N), and trabecular separation (Tb.Sp). All measurements and analyses were conducted following the guidelines set by the American Society for Bone and Mineral Research nomenclature committee.^23^

### Reverse transcription quantitative PCR analysis (RT-qPCR)

Humeri and right femora were pulverized, and total bone RNA was extracted with TRIzol Reagent (Thermo Fisher Scientific) followed by the RNeasy kit (Qiagen), according to the manufacturer’s protocol. RNA from cell cultures was extracted using the Quick-RNA Miniprep Kit (Zymoresearch). cDNA was synthesized from 140 ng RNA in a 20 μL reaction using the SuperScript VILO kit (Thermo Fisher Scientific). Target gene expression was analyzed in duplicate by quantitative real-time PCR (qPCR) with the LightCycler 480 SYBR Green I Master (Roche) and the LightCycler 480 System (Roche), according to the qPCR program detailed in Table S2. Primer sequences are listed in Table S3. Data were analyzed with the LightCycler 480 Software, version 1.5 (Roche), and normalized to TATA-box binding protein (*Tbp*). Relative gene expression was determined using the ΔΔCt method.

### RNA sequencing (RNA-Seq)

RNA extracted from the bone tissue of five hiOI and WT 8-wk-old male mice was used for RNA-Seq analysis. The Illumina TruSeq Stranded mRNA library prep kit (Illumina, Inc.) was used to prepare RNA libraries. The sequencing was performed with the NovaSeq 6000 sequencing platform (Illumina, Inc.). RNA and libraries quality and quantity were assessed using RNA Analysis ScreenTape (Agilent Technologies) and D1000 ScreenTape using 4200 TapeStation System (Agilent Technologies), respectively. RNA samples had a concentration between 58 and 67 ng/μL, with an RNA integrity number ranging between 8.7 and 9.3.

Analysis of paired-end sequencing reads was performed using the web-based platform Galaxy (https://usegalaxy.eu/). Quality control was performed with FastQC and MultiQC. The reads were mapped to the mm10/GRCm38 with HISAT2, counted with featureCounts and annotated with the annotateMyIDs. For differential expression and normalization, the limma-voom package and Heatmap were used. For gene ontology analysis, GOSeq was used. Differentially expressed genes with adjusted *p*-value lower than .05 were further analyzed with Metascape gene ontology (https://metascape.org/).

### ELISA

The serum levels of procollagen type I N-terminal propeptide (P1NP) (P1NP EIA kit, AC-33F1, Immunodiagnostic systems), a bone formation marker, were assessed in 8-wk-old and 24-wk-old mice by ELISA according to the manufacturer’s protocol. Additionally, the serum levels of tartrate-resistant acid phosphatase (TRAcP) 5b (SB-TR103, Immunodiagnostic systems) were analyzed in 8-wk-old mice to assess bone resorption.

### Collagen extraction and western blotting

Ulnae from 42-wk-old mice (3 WT and 3 hiOI) were weighed, pulverized, and demineralized in 0.5 M EDTA (46-034-CI, Corning) for 7 d at 4 °C. Following demineralization, samples were digested with 0.1 mg/mL pepsin (V195A, Promega) in 0.5 M acetic acid (pH 2.0). Collagen was precipitated by adding 4 M NaCl in 1 M acetic acid, and the resulting collagen pellet was resuspended in 0.5 M acetic acid for subsequent western blotting (WB) analysis, as described previously^24^ (Supplementary material). Band intensities for α1(I), α2(I), and total collagen type I were quantified using ImageJ software (U.S. National Institutes of Health, https://imagej.net/ij/), normalized to tissue weight and expressed as the percentage reduction in hiOI mice compared to WT.

### Human cells

#### Fibroblasts

Primary dermal human fibroblasts (Table 1) were cultured in Ham’s F-10 Nutrient Mix medium (Gibco), 10% FBS (10270-106, Gibco), and 1% penicillin/streptomycin (15140-122, Gibco), in an environment of 37 °C and 5% CO_2_.

**Table 1:** Characteristics of OI and healthy control cell lines used in the experiments.

**Cell type**	**Genotype**	**Affected gene**	**Variant**	**Sex**	**Age**
**Fibroblasts**	HI OI	*COL1A1*	c.2784delT	M	38y
**Fibroblasts**	HI OI	*COL1A1*	c.1804G>T	F	33y
**Fibroblasts**	HI OI	*COL1A1*	c.495T>A	F	29y
**Fibroblasts**	Healthy control	NA	NA	M	33y
**Fibroblasts**	Healthy control	NA	NA	F	0y
**Fibroblasts**	Healthy control	NA	NA	F	0y
**iPSCs**	Healthy control	NA	NA	M	19y
**iPSCs**	Healthy control	NA	NA	M	19y
**iPSCs**	Healthy control	NA	NA	M	19y
**iPSCs**	HI OI	*COL1A1*	c.2784delT	M	38y
**iPSCs**	HI OI	*COL1A1*	c.2784delT	M	38y
**iPSCs**	HI OI	*COL1A1*	c.2784delT	M	38y

Abbreviations: F, Female; HI OI, haploinsufficient osteogenesis imperfecta; M, male; NA, non-applicable; Y, years old.

#### Induced stem cells

The induced pluripotent stem cell (iPSC) lines were obtained from the biobank of Center for Connective Tissue Disorders, Amsterdam UMC (Table 1). Generation, validation, and maintenance of these iPSC cell lines, as well as the induction of iPSCs into iMSCs, was performed as previously described^25^ (Supplementary material).

#### Heterozygous CRISPR/Cas knockout of *COL1A1* in human induced mesenchymal stem cells

CRISPR/Cas9 editing of human healthy control iPSCs was performed to create HI of *COL1A1* via non-homologous end joining. iPSCs were dissociated from the cell culture colonies into single cells with Accutase (Stemcell Technologies). In a volume of 0.025 mL of Primary Cell P3 buffer (Lonza), 2 × 10^5^ iPSC cells were nucleofected using the Lonza Amaxa 4D nucleofector (Program CA137) with 2:1 ratio of 70 pmol Cas9 protein (Synthego) and 35 pmol dCas9 protein (Synthego), and 120 pmol single guide RNA (UCUGUACGCAGGUGAUUGGU, Synthego) designed to create a double-stranded break at the location chr17:50199941, targeting exon 2 of the *COL1A1* gene. Cas9 ribonucleoprotein complexes were assembled in vitro for 10 min at room temperature and then kept on ice. The nucleofected cells were plated into a single well of Matrigel-treated 24-well plate cultured in Essential 8 medium (A1517001, Gibco) containing 10 μM Rock Inhibitor (Selleckchem). The culture media were refreshed daily and cells were maintained until reaching confluency. Following the cloning of single-cell-derived colonies, confluent wells from replicate 96-well plates were lysed for genotyping. The target region was amplified by PCR (forward primer, 5′-GAGGAGCAGGCGAGCTTTTA-3′; reverse primer, 5′-GGGTGACTCTAGGGGACGAA-3′) and subjected to Sanger sequencing. From a screen of 96 clones, three clones with heterozygous knockout of the *COL1A1* were identified and expanded for further gene expression analysis.

### Statistical analysis

Data analysis was performed using GraphPad Prism version 9 (GraphPad Software Inc.). Normality was assessed using the Kolmogorov–Smirnov test. For normally distributed data, an unpaired *t*-test was applied. Two-way ANOVA, corrected for main effect and including multiple comparisons, was used for gender subgroups. Correlations were assessed using Pearson correlation analysis. *p*-values ≤.05 were considered statistically significant. Graphs were created with GraphPad Prism version 9 (GraphPad Software Inc.).

## Results

### Haploinsufficient OI (hiOI) mouse: a novel model for haploinsufficient OI

The targeted heterozygous deletion of 16350 bp in *Col1a1* gene, from intron 1 to 3′ UTR region (NC_000077.7: 94828227_94844576del), was introduced to create the haploinsufficient OI (hiOI) mouse line C57BL/6N-*Col1a1*^+/hiOI^ (Figure 1A). CRISPRater efficacy scores were classified as high (>0.74). The genotyping of hiOI heterozygotes shows a 765 bp band for the knockout allele and an 890 bp band for the WT allele (Figure 1B). No homozygotes were born, and only heterozygous animals were investigated during the study. At the age of 8 and 24 wk, hiOI mice were lighter compared to WT littermates (*p* ≤ .05) (Figure 1C). Femora of hiOI mice were shorter compared to WT controls at the age of 8 and 24 wk (*p* ≤ .05) (Figure 1D). Mutant mice did not differ visually from WT littermates. No skeletal dysplasia, nor spontaneous bone fractures were noticed in hiOI mice during the study. The absence of skeletal dysplasia was visually assessed, with representative CT images shown in Figure 1E.

**Figure 2:**
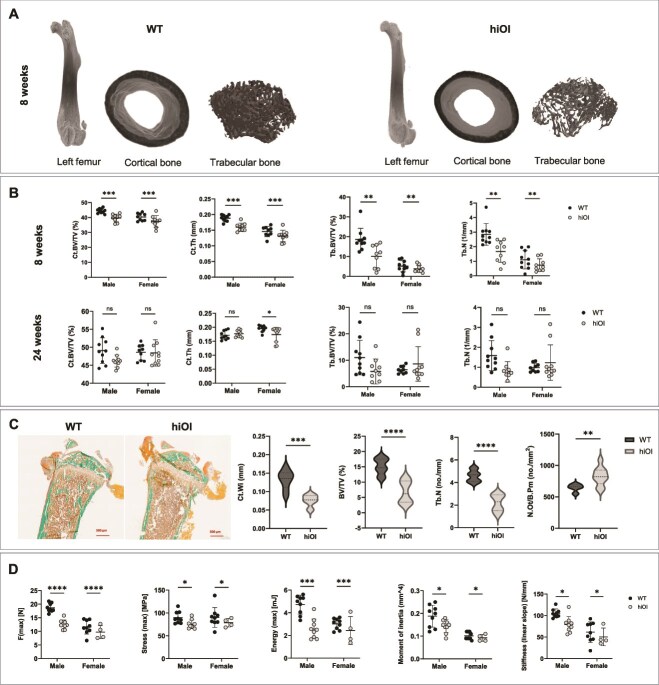
Bone phenotype of the hiOI mice. (A) Representative image of micro-CT from the left femur of 8-wk-old WT and hiOI males (whole femur, distal trabecular bone, middle shaft cortical bone). (B) Micro-CT parameters of the 8- and 24-wk-old male and female mice. Bone volume and cortical and trabecular bone parameters (Ct.BV/TV, Ct.Th, Tb.BV/TV, and Tb.N) are reduced in hiOI males and females of 8 wk; however, bone phenotype tends to be recovered at the age of 24 wk; data shown as mean ± SD. (C) Representative images of Masson–Goldner trichrome staining of 8-wk-old male mice. Histomorphometry analysis confirmed reduction in cortical width (Ct.Wi), bone to tissue ratio (BV/TV), trabecular number (Tb.N), and increase in osteocyte number (Oc.N). (D) Tree-point bending test with femora of 8-wk-old animals. Maximum force, maximum stress, maximum energy, moment of inertia, and stiffness were significantly reduced in hiOI mice. Data shown as mean ± SD. ^*^p ≤ .05, ^**^p ≤ .01, ^***^p ≤ .001, ^****^p ≤ .0001.

### The hiOI mice show reduced bone volume, altered bone microarchitecture, and decreased strength

To investigate bone microarchitecture alterations caused by *Col1a1* heterozygous knockout, we performed micro-CT analysis of mouse femora (Figure 2A). Ct.BV/TV (*p* ≤ .001), Ct.Th (*p* ≤ .001), Ct.BV (*p* ≤ .001), Ct.Ar (*p* ≤ .001), and Ct.Ar./Tt.Ar (*p* ≤ .001) were significantly reduced in 8-wk-old hiOI mice (Figure 2B, Figure S1A). At the age of 8 wk, the trabecular bone in hiOI mice also exhibited reduced Tb.BV/TV (*p* ≤ .01), Tb.N (*p* ≤ .01), Tb.BV (*p* ≤ .01), Tb.BS/TV (*p* ≤ .01), and Conn.Dn (*p* ≤ .01) (Figure 2B, Figure S1A). Tb.BS/BV, Tb.Th, and Tb.Sp did not show significant differences compared to WT littermates (Figure S1A).

**Table 2:** Differentially expressed genes in bone tissue of hiOI mice compared to WT mice.

**ENTREZID**	**Gene symbol**	**Gene name**	**logFC**	**Adjusted *p*-value**
**54388**	*H1f9*	H1.9 linker histone	6.4592	2.46E−05
**20391**	*Sgca*	Sarcoglycan, alpha (dystrophin-associated glycoprotein)	3.9887	2.46E−05
**217124**	*Ppp1r9b*	Protein phosphatase 1, regulatory subunit 9B	0.9860	8.91E−05
**407809**	*BC055402*	Cdna sequence BC055402	0.9297	.0026
**20349**	*Sema3e*	Sema domain, immunoglobulin domain (Ig), short basic domain, secreted, (semaphorin) 3E	2.0350	.0026
**71355**	*Col24a1*	Collagen, type XXIV, alpha 1	0.9433	.0075
**16367**	*Irs1*	Insulin receptor substrate 1	0.6451	.0075
**268902**	*Robo2*	Roundabout guidance receptor 2	0.7802	.0193
**226999**	*Slc9a2*	Solute carrier family 9 (sodium/hydrogen exchanger), member 2	0.9927	.0193
**12814**	*Col11a1*	Collagen, type XI, alpha 1	0.8667	.0193
**270120**	*Fat3*	FAT atypical cadherin 3	0.8438	.0193
**73173**	*Pcdh18*	Protocadherin 18	0.5443	.0193
**26432**	*Plod2*	Procollagen lysine, 2-oxoglutarate 5-dioxygenase 2	0.6647	.0193
**12815**	*Col11a2*	Collagen, type XI, alpha 2	0.8562	.0193
**13176**	*Dcc*	Deleted in colorectal carcinoma	1.4055	.0195
**13406**	*Dmp1*	Dentin matrix protein 1	0.9140	.0214
**18675**	*Phex*	Phosphate regulating endopeptidase homolog, X-linked	0.8370	.0245
**234214**	*Sorbs2*	Sorbin and SH3 domain containing 2	0.5036	.0253
**14089**	*Fap*	Fibroblast activation protein	0.5093	.0265
**18553**	*Pcsk6*	Proprotein convertase subtilisin/kexin type 6	0.8266	.0268
**13527**	*Dtna*	Dystrobrevin alpha	0.8221	.0272
**278279**	*Tmtc2*	Transmembrane and tetratricopeptide repeat containing 2	0.6793	.0311
**105005**	*Lratd1*	LRAT domain containing 1	1.2014	.0319
**12819**	*Col15a1*	Collagen, type XV, alpha 1	0.7444	.0319
**17436**	*Me1*	Malic enzyme 1, NADP(+)-dependent, cytosolic	0.6905	.0319
**67374**	*Jam2*	Junction adhesion molecule 2	0.5474	.0319
**17470**	*Cd200*	CD200 molecule	0.5195	.0319
**215819**	*Nhsl1*	NHS-like 1	0.6972	.0351
**239606**	*Slc2a13*	Solute carrier family 2 (facilitated glucose transporter), member 13	0.6585	.0403
**12832**	*Col5a2*	Collagen, type V, alpha 2	0.8727	.0403
**18552**	*Pcsk5*	Proprotein convertase subtilisin/kexin type 5	0.6482	.0403
**110075**	*Bmp3*	Bone morphogenetic protein 3	0.6560	.0403
**69700**	*Col22a1*	Collagen, type XXII, alpha 1	0.7091	.0403
**18451**	*P4ha1*	Procollagen-proline, 2-oxoglutarate 4-dioxygenase (proline 4-hydroxylase), alpha 1 polypeptide	0.6522	.0403
**12294**	*Cacna2d3*	Calcium channel, voltage-dependent, alpha2/delta subunit 3	0.9056	.0403
**20356**	*Sema5a*	Sema domain, seven thrombospondin repeats (type 1 and type 1-like), transmembrane domain (TM) and short cytoplasmic domain, (semaphorin) 5A	0.6427	.0403
**22359**	*Vldlr*	Very low density lipoprotein receptor	0.6387	.0429
**212712**	*Satb2*	Special AT-rich sequence binding protein 2	0.5470	.0429
**19876**	*Robo1*	Roundabout guidance receptor 1	0.4707	.0429
**110893**	*Slc8a3*	Solute carrier family 8 (sodium/calcium exchanger), member 3	0.6164	.0429
**12876**	*Cpe*	Carboxypeptidase E	0.5409	.0429
**140577**	*Ankrd6*	Ankyrin repeat domain 6	0.5829	.0478
**19272**	*Ptprk*	Protein tyrosine phosphatase, receptor type, K	0.5117	.0478
**17389**	*Mmp16*	Matrix metallopeptidase 16	0.5869	.0478
**14677**	*Gnai1*	Guanine nucleotide binding protein (G protein), alpha inhibiting 1	0.6599	.0478
**330286**	*D630045J12Rik*	RIKEN cdna D630045J12 gene	0.6124	.0478
**56188**	*Fxyd1*	FXYD domain-containing ion transport regulator 1	0.6148	.0478
**227377**	*Farp2*	FERM, rhogef and pleckstrin domain protein 2	0.6364	.0478
**107515**	*Lgr4*	Leucine-rich repeat-containing G protein-coupled receptor 4	0.5241	.0478
**50791**	*Magi2*	Membrane associated guanylate kinase, WW and PDZ domain containing 2	0.6467	.0492
**54216**	*Pcdh7*	Protocadherin 7	0.5622	.0492

At the age of 24 wk, cortical parameters in hiOI mice improved, and reduced Ct.Th was only noted in female mice (*p* ≤ .05). At the age of 24 wk, only Tb.Sp (*p* ≤ .05) was increased compared to WT mice. These data indicate reduced trabecular and cortical bone structure in 8-wk-old hiOI mice, which partly ameliorates at the age of 24 wk.

**Figure 3:**
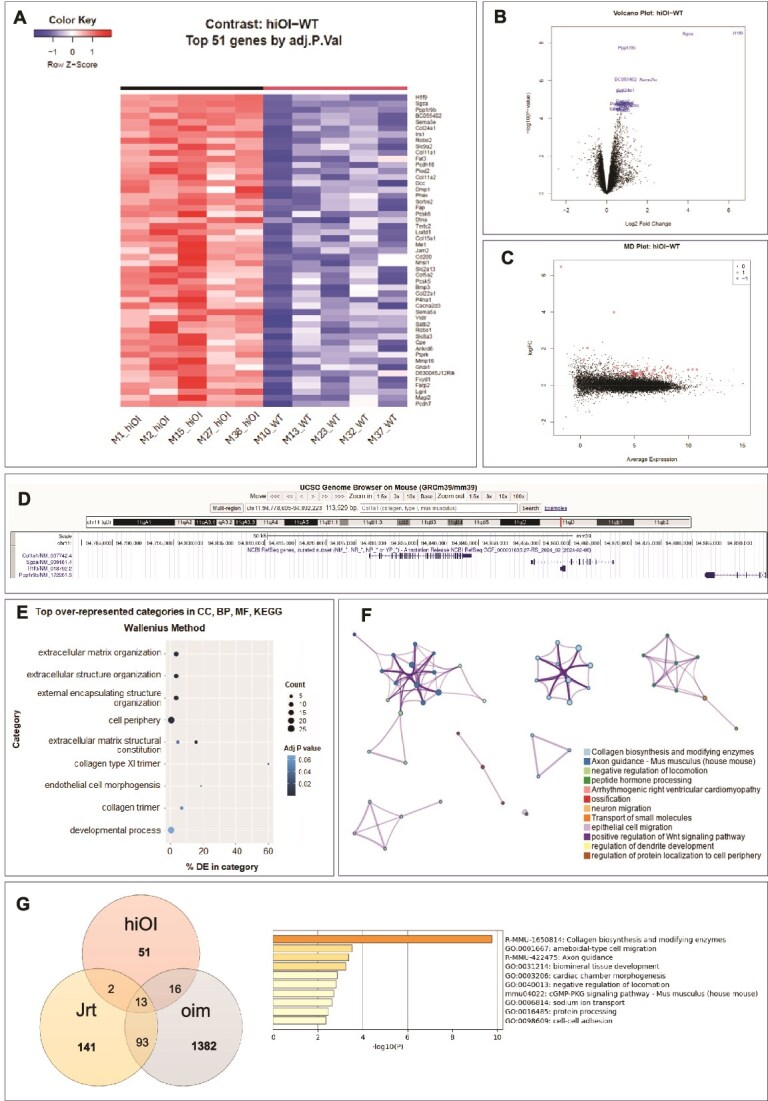
RNA sequencing analysis reveals the upregulation of 51 genes in 8-wk-old hiOI mice compared to WT mice. (A) Heatmap showing differentially expressed genes. (B) Volcano plot illustrating the upregulated genes. (C) MD plot indicating the presence of upregulated genes with no downregulated genes in hiOI mice. (D) Mouse *Col1a1* locus on chromosome 11, and the top three genes (*H1f9*, *Sgca*, *Ppp1r9b*) with the highest fold change, all located within the same genomic region. Screenshot captured from the UCSC Genome Browser (https://genome.ucsc.edu/). (E) Top over-represented Gene Ontology (GO) categories. CC, cellular component; BP, biological process; MF, molecular function, KEGG, Kyoto Encyclopedia of Genes and Genomes. (F) Network representation of the GO analysis, with each node representing a GO term. Node size is proportional to the number of genes within each term, and color denotes cluster identity. (G) Comparison of differentially expressed genes in the bone RNA of hiOI, Jrt, and oim mice. The number of upregulated genes in each mouse model compared to WT animals is shown, along with the number of upregulated genes shared across models. The enrichment of GO terms for differentially expressed genes common to OI mouse models is presented.

**Figure 4:**
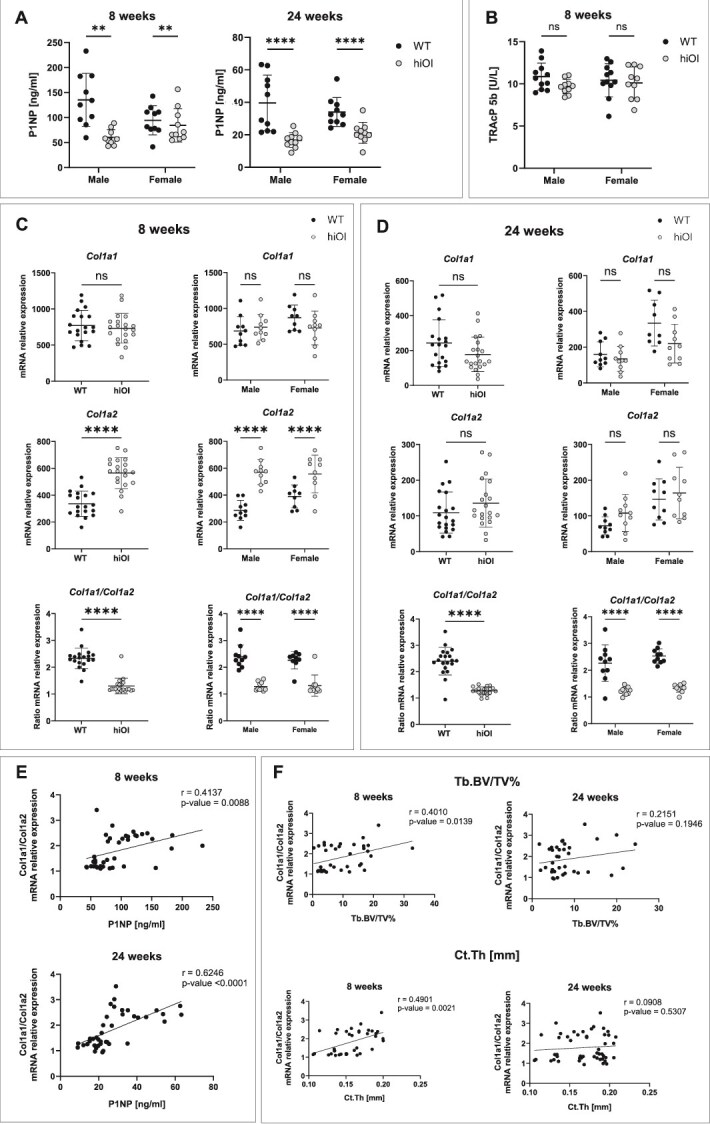
Altered collagen gene expression and serum biomarkers in hiOI mice. (A) Serum P1NP levels were significantly reduced in hiOI mice at 8 and 24 wk of age. (B) Serum TRAcP 5b levels remained normal in hiOI mice. (C-D) Real-time quantitative PCR analysis of *Col1a1* and *Col1a2* mRNA expression and their ratio in 20 hiOI and 20 WT mice at 8 wk (C) and 24 wk of age (D). (E) Serum P1NP levels were positively correlated with the *Col1a1/Col1a2* mRNA expression ratio in bone at 8 wk (*p* = .0088, *r* = 0.4137) and 24 wk (*p* < .0001, *r* = 0.6246). (F) The *Col1a1/Col1a2* ratio was correlated with trabecular and cortical bone volume in 8-wk-old mice but not in 24-wk-old mice. *p*-values are indicated as follows: ≤.05 (^*^), ≤.01 (^**^), ≤.001 (^***^), ≤.0001 (^****^). Data shown as mean ± SD. All comparisons between WT and hiOI data were performed using an unpaired *t*-test. Gender subgroups were analyzed using a two-way ANOVA with a main effect and multiple comparisons. Correlations between P1NP levels, collagen mRNA expression ratios, and bone volumes were assessed using Pearson correlation analysis.

**Figure 5:**
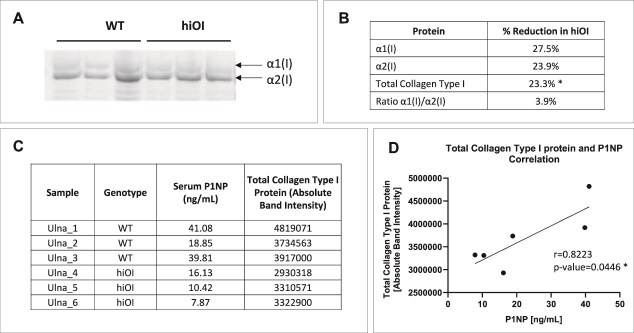
Reduced bone collagen expression in hiOI mice. (A) Western blot analysis of α1(I) and α2(I) collagen protein bands extracted from ulnae bones of WT (*n* = 3) and hiOI (*n* = 3) mice. (B) Quantification showing the percentage reduction of α1(I), α2(I), total collagen type I, and the *Col1a1*/*Col1a2* protein ratio in hiOI relative to WT ulnae. (C) Genotype, *Col1a1*/*Col1a2* mRNA ratio, serum P1NP levels, and total collagen type I (normalized to tissue weight) in ulnae. (D) A positive correlation between serum P1NP levels and total collagen type I measured by western blot is shown. Statistical significance indicated by ^*^ for *p* ≤ .05.

**Figure 6:**
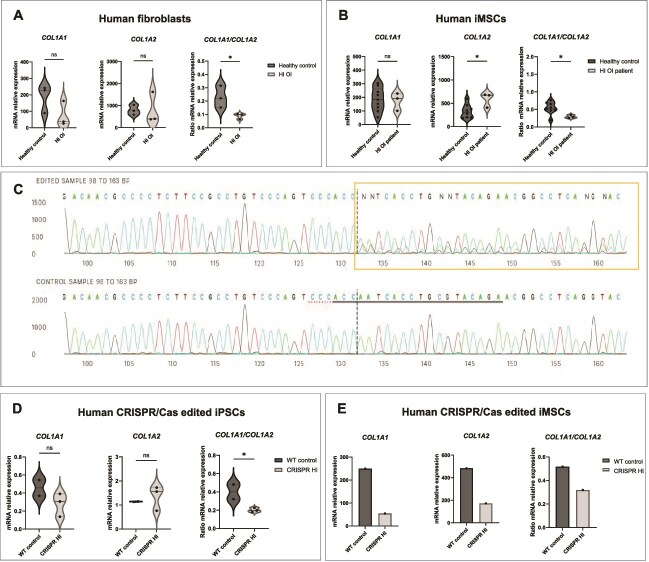
*COL1A1* and *COL1A2* expression in human HI OI fibroblasts, iMSCs, and CRISPR-edited iPSCs and iMSCs. (A and B) Real-time quantitative PCR analysis of the *COL1A1* and *COL1A2* genes and their expression ratio in human primary healthy control and HI OI patient fibroblasts (A) and iMSCs (B). (C) Sanger sequencing depicting a HI OI frameshift mutation introduced in the edited iPSC lines. (D and E) Expression of *COL1A1*, *COL1A2*, and their ratio in CRISPR/Cas-edited iPSCs (D) and iMSCs (*n* = 1) (E). *p*-values ≤.05 (^*^), ≤.01 (^**^), ≤.001 (^***^), ≤.0001 (^****^). All WT data were compared to HI OI data using an unpaired *t*-test.

These differences in histomorphometric parameters between hiOI and WT littermates were also confirmed with Masson-Goldner trichrome staining in 8-wk-old male mice (Figure 2C). Significantly lower Ct.Wi (*p* ≤ .001), Ct.BV/TV (*p* ≤ .0001), Ct.B.Pm/T.Ar (*p* ≤ .001), Tb.Wi (*p* ≤ .05), Tb.BS/TV (*p* ≤ .0001), Tb.Th (*p* ≤ .05), and Tb.N (*p* ≤ .0001) were found in hiOI mice in comparison with WT mice. Significantly higher osteocyte number (N.Ot/B.Pm) (*p* ≤ .01) and Tb.Sp (*p* ≤ .001) were found in hiOI mice. However, osteoid perimeter (O.Pm/B.Pm) did not show significant differences (Figure 2C, Figure S1C).

To evaluate bone strength, we performed three-point bending test with femora of 8-wk-old animals. The femora of hiOI mice displayed reduced stiffness (*p* ≤ .05, linear slope) and maximum force (load) (*p* ≤ .05), confirming bone weakness. Similarly, the moment of inertia (*p* ≤ .05), maximum energy (*p* ≤ .001), and maximum energy density (*p* ≤ .001) were significantly reduced in hiOI mice, validating the brittleness of the bones, and the lower energy required to induce a fracture in hiOI bone (Figure 2D, Figure S1D). Elastic modulus (linear slope), maximum strain, and maximum deformation did not differ from WT littermates (Figure S1D).

### The hiOI mice exhibit alterations in regulation of gene expression related to extracellular matrix, ossification, and osteogenesis

Bone tissue RNA-Seq of five hiOI and five WT 8-wk-old male mice revealed 51 upregulated genes in the hiOI mouse model (Table 2, Figure 3). No downregulated genes were found (Figure 3A-C). The three top upregulated genes (*H1f9*, *Sgca*, *Ppp1r9b*) were located in the same genomic region as *Col1a1* (chr11:94925000-95025000) (Figure 3D). Variability in the transcriptomes within groups of both hiOI and WT mice was observed (Figure S2). According to Gene Ontology analysis, differentially expressed genes are associated with collagen trimer biosynthesis, modifying enzymes, and extracellular matrix (ECM) development. Some of the upregulated genes were also connected to ossification, positive regulation of the Wnt signaling pathway, cell adhesion, and locomotion (Table 2, Figure 3E and F). The results of the bone RNA-Seq point toward dysregulation of the ECM and alterations in bone metabolism caused by *Col1a1* HI.

The 14 most significant upregulated genes were validated with RT-qPCR across the entire sample groups of 20 hiOI and 20 WT mice (both genders) of both 8- and 24-wk-old mice. At 8 wk of age, significant upregulation was confirmed for all genes, in both gender subgroups, apart from *Irs1* in females (Figure S3A). At 24 wk of age, only the *H1f9*, *Sgca*, and *Dcc* genes showed upregulation compared to WT animals, in agreement with the improvement in bone phenotype (Figure S3B).

### The hiOI mice have reduced levels of P1NP

At the age of 8 wk, hiOI mice showed a 40.83% decrease in the amount of serum P1NP (*p* ≤ .001) compared to WT mice (67.98 ± 20.71 ng/mL and 114.90 ± 46.61 ng/mL, respectively). At 24 wk, hiOI mice had 48.57% reduction in serum P1NP (*p* ≤ .0001; 18.95 ± 5.96 ng/mL and 36.85 ± 13.58 ng/mL, respectively). Differences were also significant in both gender subgroups for both age groups (Figure 4A). P1NP is a collagen type I formation marker indicative of bone formation and confirms approximately a 50% reduction in collagen type I levels in hiOI mouse bones. The amount of TRAcP 5b did not differ between WT and hiOI animals, implicating collagen type I insufficiency, and not bone resorption, as the origin of the reduced bone volume and altered microarchitecture (Figure 4B).

### The hiOI mouse model shows dysregulated mRNA ratio between the collagen type I genes

In contrast to our expectations, the *Col1a1* knockout mice did not show significant reduction in the *Col1a1* gene expression despite the heterozygous deletion of the intron 1 to 3′ UTR region of the gene (Figures 1A and 4C, D). Interestingly, a 1.7-fold increase in *Col1a2* mRNA expression in hiOI mice compared to WT was observed at 8 wk (*p* ≤ .0001) in both genders (Figure 4C), however not in 24-wk-old mice (Figure 4D). High variability in *Col1a1* and *Col1a2* gene expression can be appreciated in both hiOI and WT mice. At both timepoints and across genders, *Col1a1/Col1a2* mRNA ratio was significantly reduced (*p* ≤ .0001) (Figure 4C and D), namely by 44.33% at 8 wk and 46.83% at 24 wk (Table S4). The reduced *Col1a1/Col1a2* mRNA ratio correlated with decreased P1NP serum levels at both timepoints: 8 wk (*p* = .0088, *r* = 0.4137) and 24 wk (*p* ≤ .0001, *r* = 0.6246) (Figure 4E).

Additionally, the decreased *Col1a1/Col1a2* mRNA ratio correlated with the reduced bone volume of trabecular (*p* ≤ .05, *r* = 0.4010) and cortical (*p* ≤ .01, *r* = 0.4217) bone in 8-wk-old mice. However, this correlation was not significant in 24-wk-old animals (Figure 4F).

### Decreased bone collagen expression in hiOI mice

Western blotting analysis of ulnae (Figure 5A) showed significant decreases in collagen type I chains and total collagen type I. Levels of α1(I) collagen were reduced by 27.5%, α2(I) collagen by 23.9%, and total collagen by 23.3% (*p* = .05) in hiOI mice. The α1(I)/α2(I) protein ratio in hiOI mice was slightly decreased (3.9%) (Figure 5B). A significant positive correlation (*p* = .0446, *r* = 0.8223) was found between serum P1NP and total collagen type I measured by WB (Figure 5C and D), indicating a link between secreted collagen type I in serum and bone collagen content.

### Collagen type I gene expression and protein alterations in HI OI patient-derived cells and hiOI mouse cells

In order to test if *COL1A1/COL1A2* mRNA ratio is also altered in human HI OI patients, we analyzed the expression of the collagen type I genes in primary skin fibroblasts of patients with confirmed HI pathogenic variants (Table 1). *COL1A1* expression only showed a trend toward reduction, which was not statistically significant due to donor variability, whereas *COL1A2* exhibited a nonsignificant upward trend (Figure 6A). However, this translated to a significant *COL1A1/COL1A2* reduction compared to the healthy controls (*p* ≤ .05). This is in line with the decreased collagen type I genes expression pattern found in the bones of hiOI mice (Figure 4C, D and 6A). The same trend was found in HI OI iMSCs, with *COL1A2* expression increase and *COL1A1/COL1A2 mRNA* ratio reduction being significant (*p* ≤ .05) (Figure 6B).

In addition, we edited a healthy control cell line of iPSCs with CRISPR/Cas to create the heterozygous frameshift mutation NM_000088.4: c.111_117del p.(Ile38Alafs34^*^), which is predicted to cause a premature stop codon and nonsense-mediated mRNA decay, resulting in HI (Figure 6C). This pathogenic variant is also reported in the LOVD OI variant database as being associated with OI type 1.^26^ In agreement with findings in hiOI mice and OI patient cells, analysis of the collagen type I genes expression revealed no significant reduction in *COL1A1* expression, as well as no significant difference in *COL1A2* expression, which however resulted in significant decrease of the *COL1A1*/*COL1A2* mRNA expression ratio in the gene-edited iPSCs (Figure 6D). The described mRNA ratio differences were also observed after induction of the edited iPSC lines into iMSCs (*n* = 1) (Figure 6E), and in BMSCs derived from WT and hiOI mice (Figure S4A), supporting the *COL1A1/COL1A2* mRNA ratio as an important component of the HI OI pathomechanism.

Expression analysis of *Col1a1* locus genes in human cell models, as well as mouse BMSCs, demonstrated consistent overexpression of the *Col1a1* genomic region (Figure S4), in agreement with observations in hiOI mouse bones (Figure S3A). Statistically significant differences were identified for *SGCA* in iMSCs (*p* = .0366) (Figure S4D), and for *H1f9* (*p* = .0362) and *Sgca* (*p* = .0409) in BMSCs (Figure S4A), alongside an overall trend of increased expression in HI OI fibroblasts (Figure S4C). Western blotting analysis of collagen type I chains in BMSCs revealed a reduction in α1(I) and α2(I) protein levels in hiOI mice (Figure S5). In the medium fraction, α1(I) and α2(I) levels were reduced by 25.4% and 46.0% on average, respectively (Figure S5A and C). In the cytosolic fraction, α1(I) and α2(I) showed an ~25% decrease (Figure S5B and C). Total collagen type I levels were consistently lower in hiOI, with reductions of 39.9% in the medium and 23.0% in the cytosol. The α1(I)/α2(I) protein ratio decreased by 19.4% in the cytosol of hiOI, while it remained nearly unchanged in the medium (Figure S5C).

## Discussion

Haploinsufficient OI type 1 is often considered the mildest form of the disease, as patients typically do not exhibit skeletal dysplasia.^10^^,^^14^^,^^15^ However, this does not classify HI OI type 1 as a mild disease: patients with HI OI still experience an almost 100-fold increase in fracture rate compared to the general population, with the majority of fractures attributable to trauma, such as falls and collisions.^15^ In addition to long bone fractures, these individuals also suffer from vertebral fractures, pain, fatigue, diverse extraskeletal problems, mental health issues, and the consequences of an invisible disability, all of which significantly impact their quality of life.^27^

To provide a reliable platform for studying the molecular and physiological characteristics specific to HI OI type 1, and to facilitate the development of targeted therapies, we developed a genetically modified mouse model harboring a heterozygous allele of *Col1a1* that replicates the collagen type I HI observed in OI type 1. This novel model, named the hiOI mouse model, was created by a heterozygous deletion from intron 1 to the 3′ UTR region of the *Col1a1* gene in the C57BL/6N strain using the CRISPR/Cas editing system.

The hiOI mice, in contrast to mouse models of severe OI, do not exhibit major skeletal malformations or fractures. However, they show decreased body weight, as well as impaired bone microarchitecture and mechanical properties at 8 wk of age, confirming the presence of mild bone fragility.^16^ At 24 wk, the hiOI mice still exhibit collagen type I HI, although the bone phenotype ameliorates. Reduction in bone fragility during adulthood has also been reported in untreated human patients and other murine OI models, with fracture rates improving after puberty.^20^^,^^28^^,^^29^ As they age, the bone phenotype of Mov13 and hiOI mice partly recovers at 15 and 24 wk, respectively, albeit to a lesser extent in the Col1a1^±365^ mice. This may result from an ECM adaptation to the fragility caused by collagen type I variants,^29^ or a decrease in bone resorption,^28^ which may explain the improvement in phenotype with age in hiOI mice, despite the P1NP levels remaining decreased. Notably, collagen levels no longer significantly correlate with micro-CT indices at 24 wk. Furthermore, the pattern of differentially expressed genes identified in young mice is not observed in adult mice. Genes involved in collagen post-translational processing, such as *Crtap* and *Fkbp10*, are upregulated in 8-wk-old hiOI bone tissue, but normalize at 24 wk. However, their expression patterns in mouse BMSCs, human fibroblasts, and human iMSCs are variable (Figure S6). At 8 wk, the mice are in a phase of rapid growth, during which upregulation of genes involved in collagen synthesis and ECM formation may likely serve as a compensatory mechanism for defective collagen production. By 24 wk, however, the ECM reaches a more stable and mature state, resulting in the normalization of gene expression and possible improvement in phenotype (Table S3B). It can be speculated that, in addition to the normalization of the observed bone gene expression, other factors, such as changes in bone resorption, may play a role in the increase in bone mass observed in adult OI animals.

In contrast to Col1a1^±365^ and Col1a1^+/−^ mice, which share exon 2 to exon 5 knockout, changes in osteoclast number were not found in young hiOI and Mov13 mice.^16–19^ Increased osteoclast number and activity are typically attributed to OI defects of abnormal collagen structure, which directly affects osteoclast formation and function. However, human patients with mild OI and a quantitative defect in collagen present less evidence of osteoclast dysregulation similarly to hiOI and Mov13 mice with HI.^30^

As a consequence of the *Col1a1* knockout, serum P1NP levels are nearly reduced to half in hiOI mice, which is consistent with the Mov13 and Col1a1^±365^ models, as well as human patients.^16^^,^^19^^,^^31^^,^^32^ A reduction in α1(I), α2(I), and total collagen type I protein was observed in hiOI bone tissue, as well as in the medium and cytosol of BMSCs derived from hiOI mice, confirming collagen type I HI in this model.

Transcriptome analysis of the hiOI mice revealed alterations not only in collagen biosynthesis and ECM regulation gene networks, but also in Wnt signaling and ossification. The Wnt signaling pathway plays a key role in bone metabolism, and defects in genes involved in the pathway are known to cause various bone fragility disorders. Interestingly, homozygous pathogenic variants in the *WNT1* are associated with moderate-severe OI, whereas heterozygous variants cause early-onset osteoporosis.^33^^,^^34^ Furthermore, genes associated with or regulated by the TGF-β signaling pathway, such as *Bmp3 and Plod2*, are significantly upregulated. The TGF-β pathway is critically involved in ECM organization and modulation of the cell periphery, both of which are significantly enriched categories in our transcriptome data. Comparison of the upregulated genes in hiOI mice with those identified in transcriptome analyses of other mouse models revealed fewer dysregulated genes in hiOI mice (51), compared to Jrt (231), oim (2969),^35^ and Col1a1^+/−^ (2086)^18^ mice. Out of the 51 genes, 31 were shared with Jrt (2), oim (16), or both (13) OI models (Figure 3G; Table 3). Although Jrt (*Col1a1*) and oim (*Col1a2*) present structural collagen abnormalities, they share upregulation of genes with the HI OI mouse model related to collagen biosynthesis and modification as well as biomineral tissue development. These findings are additionally supported by RNA sequencing data from Col1a1^+/−^ mice, where upregulation of the Wnt and TGF-β signaling pathways was also observed, consistent with our results.^18^

**Table 3:** Upregulated genes shared between hiOI, Jrt, and oim mouse models.

**#**	**Gene**	**OI mouse models**
**1**	*Slc9a2*	hiOI, Jrt
**2**	*Tmtc2*	hiOI, Jrt
**3**	*Bmp3*	hiOI, Jrt, oim
**4**	*Col11a1*	hiOI, Jrt, oim
**5**	*Col11a2*	hiOI, Jrt, oim
**6**	*Col22a1*	hiOI, Jrt, oim
**7**	*Col24a1*	hiOI, Jrt, oim
**8**	*Cpe*	hiOI, Jrt, oim
**9**	*Dmp1*	hiOI, Jrt, oim
**10**	*Fap*	hiOI, Jrt, oim
**11**	*Farp2*	hiOI, Jrt, oim
**12**	*Pcdh7*	hiOI, Jrt, oim
**13**	*Pcsk6*	hiOI, Jrt, oim
**14**	*Phex*	hiOI, Jrt, oim
**15**	*Plod2*	hiOI, Jrt, oim
**16**	*Cd200*	hiOI, oim
**17**	*Col5a2*	hiOI, oim
**18**	*Dtna*	hiOI, oim
**19**	*Fxyd1*	hiOI, oim
**20**	*Gnai1*	hiOI, oim
**21**	*Irs1*	hiOI, oim
**22**	*Jam2*	hiOI, oim
**23**	*Lgr4*	hiOI, oim
**24**	*Me1*	hiOI, oim
**25**	*P4ha1*	hiOI, oim
**26**	*Pcdh18*	hiOI, oim
**27**	*Ptprk*	hiOI, oim
**28**	*Robo1*	hiOI, oim
**29**	*Sema5a*	hiOI, oim
**30**	*Slc8a3*	hiOI, oim
**31**	*Vldlr*	hiOI, oim

Interestingly, in Mov13 mice, similarly to hiOI, we did not observe a reduction in *Col1a1* mRNA expression in the bone tissue.^16^ However, findings in Mov13 mice are unreliable, as gene expression changes may result from their precancerous state or alterations in intron 1, which regulates tissue-specific expression of *Col1a1*. Despite the absence of reduced *Col1a1* expression, a mild bone fragility phenotype was still present in Mov13 mice. It can be suggested that the unchanged levels of *Col1a1* expression in HI heterozygous mice result from compensatory overexpression of the WT allele. Interestingly, RNA-Seq of hiOI mouse bone tissue revealed a remarkable six-fold upregulation of genes—*H1f9*, *Sgca*, *Ppp1r9b* (UniProtKB: Q9QYL0, P82350, and Q6R891, respectively)—located in the same genomic region as *Col1a1*. Although not directly known to be involved in bone-related pathways, their upregulation occurs as a result of the increased expression of the remaining WT *Col1a1* allele. Notably, their increased expression persists at 24 wk, whereas other upregulated genes normalize by adulthood. Regulatory sequences in the deleted fragment were excluded to prevent *Col1a1* translocation. However, the observed upregulation of genes in the *Col1a1* genomic location may point to regulatory mechanisms influencing the entire locus.

Moreover, increased mRNA expression of *Col1a2* was observed in hiOI mice, similarly to Mov13.^16^ The expression of *COL1A1* and *COL1A2* is tightly coordinated to maintain a 2:1 ratio of pro-α1(I) to pro-α2(I) chains, which is necessary for the proper assembly of the collagen triple helix. This coordination is achieved through a complex interplay of transcriptional, post-transcriptional, and epigenetic mechanisms to ensure the proper synthesis and assembly of collagen type I.^3^^,^^36^^,^^37^ Shared regulatory elements and transcription factors, such as Specificity protein 1 (Sp1), CCAAT-binding transcription factor, and TGF-β, can influence the transcription of both genes.^38–41^ The stability and translation of *COL1A1* and *COL1A2* mRNAs can be regulated by microRNAs and RNA-binding proteins, further modulating collagen production.^39^ Additionally, regulatory feedback loops may exist where *COL1A1* influences *COL1A2* expression, and imbalances in chain composition might trigger cellular responses to adjust transcription levels to maintain proper collagen assembly.^3^^,^^37^ In contrast to hiOI and Mov13, the Col1a1^+/−^ mice show a decrease in *Col1a1* mRNA expression; however, data on *Col1a2* expression are unavailable, preventing conclusions about the collagen mRNA ratio in this model.

As observed in hiOI mice, Mov13 mice, human primary HI OI and *COL1A1* heterozygous knockout-edited cells, the decrease in the *COL1A1/COL1A2* mRNA ratio corresponds to reduced type I collagen, leading to HI OI and highlighting the necessity of a precise 2:1 ratio regulation for maintaining the structural integrity of connective tissues.^40^^,^^42^ In the case of Mov13 mice, this previously presented lack of clarity due to the mice suffering from severe leukemia at the investigated age. Importantly, the reduced *COL1A1/COL1A2* mRNA ratio is associated with HI OI, particularly in cases involving frameshift, nonsense, and specific splice-site variants of *COL1A1*. In contrast, glycine substitution variants, which primarily impair collagen structure rather than quantity, may not be linked to the collagen type I mRNA ratio decrease.

Collagen type I is a dosage-sensitive protein: its absence results in lethality, reduced expression leads to the development of HI OI type 1, and excessive production of individual collagen type I chains causes the formation of homotrimers, fibrosis and pathological states.^39^^,^^43–45^ Molecules containing pro-α2(I) chains only are not assembled.^46^ The ratio of pro-α1(I) to pro-α2(I) chains in the procollagen remains unchanged. However, free pro-α2(I) chains can undergo intracellular degradation, which limits procollagen I synthesis by restricting production to the availability of pro-α1(I) chains, as shown in OI type 1 fibroblasts by Barsh et al.^46^ Notably, mature collagen type I is located in the ECM.^5^ Consequently, at the protein level, changes in the α1(I) to α2(I) ratio are not expected in the ECM despite reduced collagen type I production, since collagen molecules in the ECM are fully assembled and maintain their 2:1 stoichiometry. This likely accounts for the minimal change in the α1(I)/α2(I) protein ratio observed in both BMSC-conditioned medium and bone tissue, in contrast to a more pronounced decrease (approximately 20%) seen in the cytosolic fraction of BMSCs. Similarly, the *COL1A1* and *COL1A2* genes are dosage-sensitive. In contrast to *COL1A1*, heterozygous defects predicted to reduce *COL1A2* expression do not lead to a disease phenotype; however, homozygous deletions of *COL1A2* cause cardiac valvular Ehlers–Danlos syndrome.^47^^,^^48^ Regarding *COL1A1*, cases of complete absence of gene expression are not viable.^49^ Further understanding of the HI OI mechanism underlined by dosage-sensitivity of the *COL1A1/COL1A2* ratio and *COL1A1* regulation is pivotal for development of OI collagen-targeting therapies.^50^

The hiOI mice exhibit key features of HI OI type 1, including reduced bone mass, altered bone microarchitecture and strength, decreased collagen type I protein expression, and dysregulated gene expression landscape. These characteristics validate this model as a valuable tool for accelerating the understanding of the pathophysiology of HI OI and the testing of potential therapeutic interventions for this most prevalent but under-researched form of OI. Additionally, we demonstrate for the first time that *COL1A1* mRNA expression by itself is not indicative of HI OI and that the pro-α1(I) and pro-α2(I) chain mRNA ratio must always be considered when examining the underlying mechanisms of HI OI.

## Supplementary Material

Supplementary_material_zjaf138

## Data Availability

The data will be made available upon reasonable request to the corresponding author.
