# Supplementary material for: New lens on congenital mild bone fragility: a novel Col1a1 knockout mouse model for osteogenesis imperfecta type 1
Source: J Bone Miner Res. 2025 Oct 6;41(5):565–80. doi: 10.1093/jbmr/zjaf138 (PMC13135081; doi:10.1093/jbmr/zjaf138)
Supplement: Supplementary_material_zjaf138 [file supplementary_material_zjaf138.pdf]

Journal of Bone and Mineral Research

Supplementary material

A New Lens On Congenital Mild Bone Fragility: *Col1a1* Knockout hiOI Mouse as a Novel Model for Haploinsufficient Osteogenesis Imperfecta Type 1

Lidiia Zhytnik[#1,2,3,4,5], Laura Ventura[#2,3,4,5], Anastasia Sclocco[2], Matthjis Verhage[6], Astrid D. Bakker[7], Jae-Hyuck Shim[8], Wissam Beaino[9,10], Pedro M. Pereira[9,10], Myrthe E. Hoogeland[11], Vivi M. Heine[12,13], Huub Maas[5,14], Richard T. Jaspers[5,14], Anja Niehoff[15,16], Frank Zaucke[17], Vivian de Waard[11], E. Marelise W. Eekhoff[3,4,5,18], Dimitra Micha*[2,3,4,5]

[1]*Department of Traumatology and Orthopaedics, The University of Tartu, 50410 Tartu, Estonia*
[2]*Department of Human Genetics, Amsterdam UMC location VUmc, 1081 HZ Amsterdam, The Netherlands*
[3]*Rare Bone Disease Center Amsterdam, 1081 HV Amsterdam, The Netherlands*
[4]*Amsterdam Reproduction and Development, Amsterdam, The Netherlands*
[5]*Amsterdam Movement Sciences, Amsterdam, The Netherlands*
[6]*Department of Human Genetics, Center for Neurogenomics and Cognitive Research, University Medical Center Amsterdam, 1081 HV Amsterdam, The Netherlands*
[7]*Department of Oral Cell Biology, Academic Centre for Dentistry Amsterdam (ACTA), University of Amsterdam and Vrije Universiteit Amsterdam, Gustav Mahlerlaan 3004, 1081 LA Amsterdam, The Netherlands*
[8]*Department of Cellular and Genetic Medicine, Horae Gene Therapy Center, University of Massachusetts Medical School, 01655 Worcester, MA, USA*
[9]*Department of Radiology and Nuclear Medicine, UMC location, Vrije Universiteit Amsterdam, De Boelelaan 1117, 1081 HV Amsterdam, The Netherlands*
[10]*Amsterdam Neuroscience, Brain Imaging, Amsterdam, The Netherlands*
[11]*Department of Medical Biochemistry, Amsterdam UMC location University of Amsterdam, Amsterdam Cardiovascular Sciences, Atherosclerosis & Ischaemic Syndromes, 1105 AZ Amsterdam, The Netherlands*
[12]*Department of Child and Adolescent Psychiatry, Amsterdam UMC Location Vrije Universiteit Amsterdam, Amsterdam Neuroscience, 1081 HV Amsterdam, The Netherlands*
[13]*Department of Complex Trait Genetics, Center for Neurogenomics and Cognitive Research, Vrije Universiteit Amsterdam, Amsterdam Neuroscience, 1081 HV Amsterdam, The Netherland*
[14]*Department of Human Movement Science, Faculty of Behavioral and Movement Sciences, Vrije Universiteit Amsterdam, 1081 HZ Amsterdam, The Netherlands*
[15]*Institute of Biomechanics and Orthopaedics, German Sport University Cologne, 50933 Köln, Germany*
[16]*Cologne Center for Musculoskeletal Biomechanics, Faculty of Medicine and University Hospital Cologne, University of Cologne, 50931 Köln, Germany*
[17]*Dr. Rolf M. Schwiete Research Unit for Osteoarthritis, Department of Trauma Surgery and Orthopaedics, University Hospital, Goethe University Frankfurt, 60528 Frankfurt am Main, Germany*
[18]*Department of Endocrinology and Metabolism, Amsterdam UMC location Vrije Universiteit, 1081 HV Amsterdam, The Netherlands*

[#]These authors contributed equally to this work: Lidiia Zhytnik, Laura Ventura.

* Corresponding author:
Dimitra Micha
Department of Human Genetics, Amsterdam UMC Location VUmc, De Boelelaan 1108, 1081 HZ Amsterdam, The Netherlands
d.micha@amsterdamumc.nl
tel: +31(0)20-4444444
fax: +31(0)20-4444444

Supplementary Material and Methods

hiOI mice genotyping

PCR amplification of the target region produced distinct banding patterns corresponding to each genotype. WT samples exhibited a single band at 890 bp, amplifying WT sequence in the 3'UTR region using primers F2 and R1 (Fig. 1A). Homozygous mutants, expected to produce a single band at 765 bp, were not observed due to lethality. A mutant amplicon spanning intron 1 through the 3' UTR, crossing the deleted *Col1a1* locus, was generated with primers F1 and R1. Heterozygous samples showed two bands, at 765 bp and 890 bp, consistent with the presence of both mutant and WT alleles (Fig. 1A).

Induced human cell lines

iPSCs were cultured in a humidified atmosphere at 37°C with 5% CO_2 and 5% O_2 in Essential 8 medium (A1517001, Gibco, Grand Island, NY, USA) supplemented with 1% penicillin/streptomycin (15140-122, Gibco, Grand Island, NY, USA) on matrigel-coated 6 well-plates (VWR, 657185). The medium was replaced daily, and cells were passaged once a week using Gentle Cell Dissociation reagent (100-0485, StemCell Technologies, Vancouver, CA). Induction of iPSCs into induced mesenchymal stem cells (iMSCs) via induced neural crest cells (iNCC) stage was performed. iMSCs were cultured on plastic in T75 flasks (Nunc, 156499), using α-MEM+GlutaMAX (32561-029, Gibco, Grand Island, NY, USA) medium supplemented with 10% FBS (10270-106, Gibco, Grand Island, NY, USA) and 1% penicillin/streptomycin (15140-122, Gibco, Grand Island, NY, USA), in an environment of 37°C and 5% CO_2. The medium was refreshed twice a week and cells were split once they reached 80% confluency.

Collagen Western Blotting (WB)

WB was performed using NuPAGE™ Tris-Acetate gels (EA03755BOX, Invitrogen™, Waltham, MA, USA) and NuPAGE™ Tris-Acetate SDS Running Buffer (LA0041, Invitrogen™). The nitrocellulose membrane was incubated with primary antibodies specific to collagen type I chains (C7510-17K-200ug, US Biological, Salem, MA, USA).

Isolation and culture of mouse Bone Marrow Stromal Cells (BMSCs)

BMSCs were isolated from 8-week-old hiOI (n=6) and WT mice (n=5). The proximal ends of right femur and both humeri were excised and the bones were centrifuged at 6000×g for 30 seconds to flush out the bone marrow. The bone marrow pellet was resuspended in high-glucose Dulbecco's Modified Eagle Medium (DMEM, 11965092, Gibco™, Grand Island, NY, USA) and filtered through a 100 µm cell strainer to remove bone fragments and debris. The resulting cell suspension was cultured in high-glucose DMEM, 15% FBS (10270-106, Gibco, Grand Island, NY, USA), 1% penicillin/streptomycin (15140-122, Gibco, Grand Island, NY, USA) and 40 mg/L L-Proline (P5607, Sigma-Aldrich, St. Louis, MO, USA), and incubated at 37°C in a humidified atmosphere with 5% CO_2.

BMSCs were plated on 6 well-plates for Western Blot analysis and cultured in high-glucose DMEM, 15% FBS, 1% penicillin/streptomycin and 40 mg/L L-Proline until. 24 hours before harvesting, the medium

was changed to fresh medium containing reduced FBS (5%) and added with 50 ug/mL 2-phospho-L-ascorbic acid trisodium (Sigma, Burlington, MA, USA, 49752-10G). The cells were counted with Neubauer chamber.

Western blotting for FKBP65 and HSP47 in BMSCs

For investigation of FKBP65 and HSP47, the cell pellet was resuspended in 10x NuPAGE reducing agent (Invitrogen, Waltham, MA, USA; NP0004) and 4x NuPAGE LDS sample buffer (Invitrogen, NP0007), then heated at 95°C for 10 minutes. Samples were loaded onto NuPAGE™ Bis-Tris Mini Protein Gels (4–12%, Invitrogen, NP0323BOX) and run using NuPAGE MOPS Running Buffer (20x, Invitrogen, NP0001) for approximately 70 minutes. SeeBlue Plus2 Prestained Standard (Invitrogen, LC5925) was used as a molecular weight ladder. Proteins were transferred to nitrocellulose membranes using the iBlot Transfer Stack Regular Kit (Invitrogen, IB301001). Membranes were blocked with Odyssey Blocking Buffer (LI-COR Biosciences, 927-40000) and incubated with primary antibodies overnight at 4°C, followed by secondary antibody incubation for 45 minutes at room temperature in the dark. The primary antibodies used were FKBP65 (65 kDa; 610648; BD Biosciences) and HSP47 (47 kDa; ab109117; Abcam). Actin was used as a loading control (ab190301; ab1801; Abcam). Protein bands were visualized using the Li-Cor Odyssey IR Gel Scanner (LI-COR Biosciences) and Odyssey V3.0 software. Band intensities were quantified using ImageJ software (U.S. National Institutes of Health, Bethesda, Maryland, USA; https://imagej.net/ij/) and normalized to Actin.

Collagen extraction and Western blotting for Collagen Type I in BMSCs

Collagen was isolated from both the culture media and cell fractions of BMSCs derived from 8-week-old mice.

Isolation of protein from culture medium

To 1 mL of culture medium, 20 µL 0.5 M EDTA pH 8.0 (46-034-CI, Corning Life Sciences, Corning, NY, USA), 10 µL of 5 mg/mL N-Ethylmaleimide (NEM) (34115, Merck-Millipore, Darmstadt, Germany) and 10 µL 12 mg/mL Pefabloc SC-Protease Inhibitor (11429868001, Merck, Darmstadt, Germany) were added. After ethanol precipitation, the collagen pellet was resuspended in 0.5 M acetic acid.

Isolation of protein from cytosol fraction

The cell pellet was resuspended in 200 µL of 0.5% Triton X-100 solution to induce collagen precipitation and placed at 4°C for 2 hours. After incubation, the tubes were centrifuged at maximum speed for 5 minutes.

Pepsin digestion

Samples were digested with 0.2 mg/mL pepsin in 0.5 M acetic acid, and pepsin activity was inhibited using 100 µM Pepstatin A (26305-03-3, Carl Roth, Karlsruhe, Germany).

Western blotting

Samples were diluted in 10x NuPAGE reducing agent (Invitrogen, Waltham, MA, USA; NP0004) and 4x NuPAGE LDS sample buffer (Invitrogen, NP0007), then heated at 60°C for 20 minutes. Samples were loaded onto NuPAGE™ 3–8% Tris-Acetate gels (Invitrogen, EA03755BOX) and run using NuPAGE TA-

SDS Running Buffer (20x) (Invitrogen, LA0041) for approximately 60 minutes. HiMark Pre-stained Molecular Weight Protein Standard (Invitrogen, LC5699) was used as a molecular weight ladder. Proteins were transferred to nitrocellulose membranes using the iBlot Transfer Stack Regular Kit (Invitrogen, IB301001). Membranes were blocked with Odyssey Blocking Buffer (LI-COR Biosciences, 927-40000) and incubated with primary antibodies specific to collagen type I chains (C7510-17K-200ug, US Biological, Salem, MA, USA) overnight at 4°C. Protein bands were visualized using the Li-Cor Odyssey IR Gel Scanner (LI-COR Biosciences) and Odyssey V3.0 software. Band intensities were quantified using ImageJ software (U.S. National Institutes of Health, Bethesda, Maryland, USA; https://imagej.net/ij/) and normalized to the cell count.

Supplementary Figures

Fig. S1

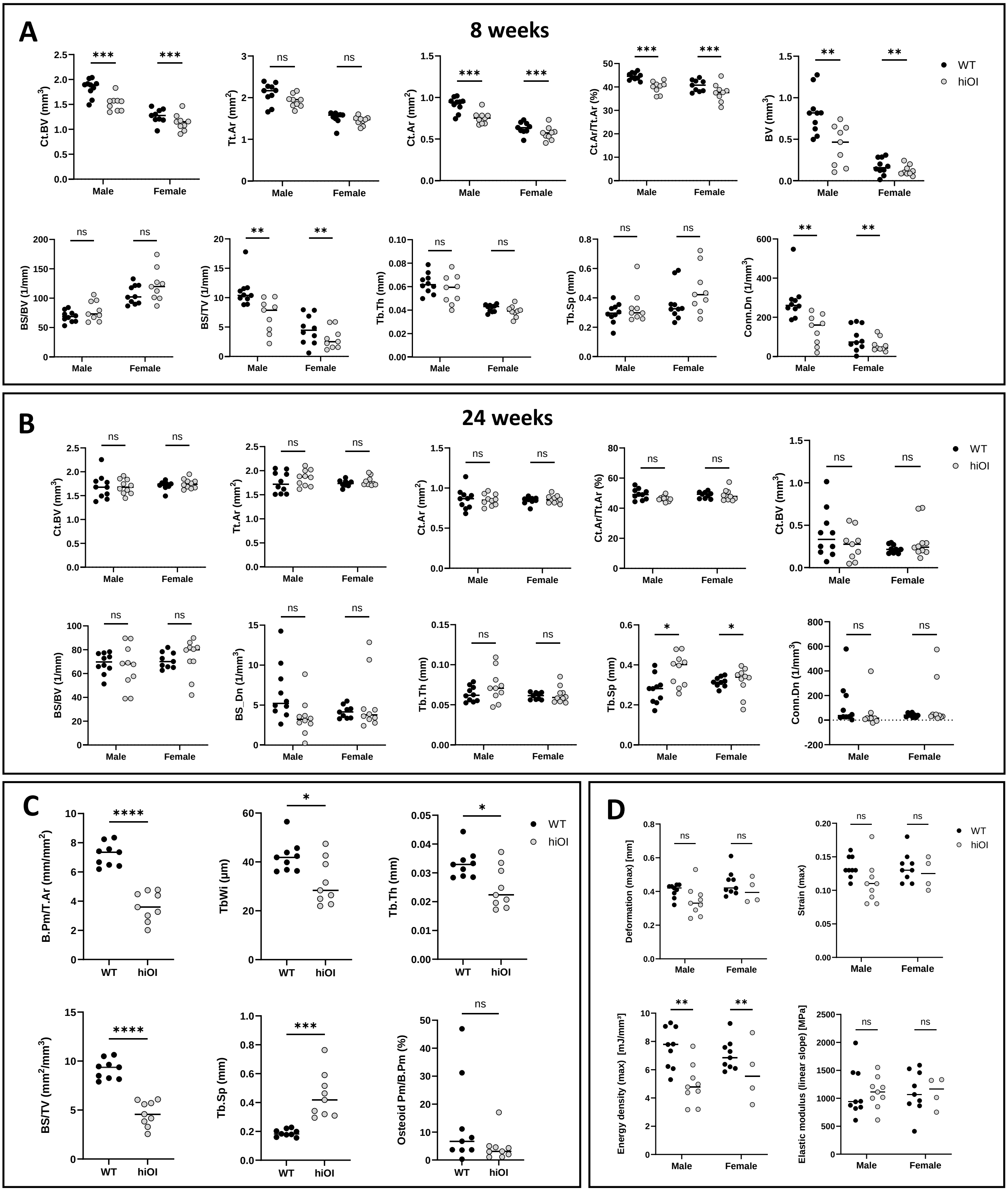

Fig. S2

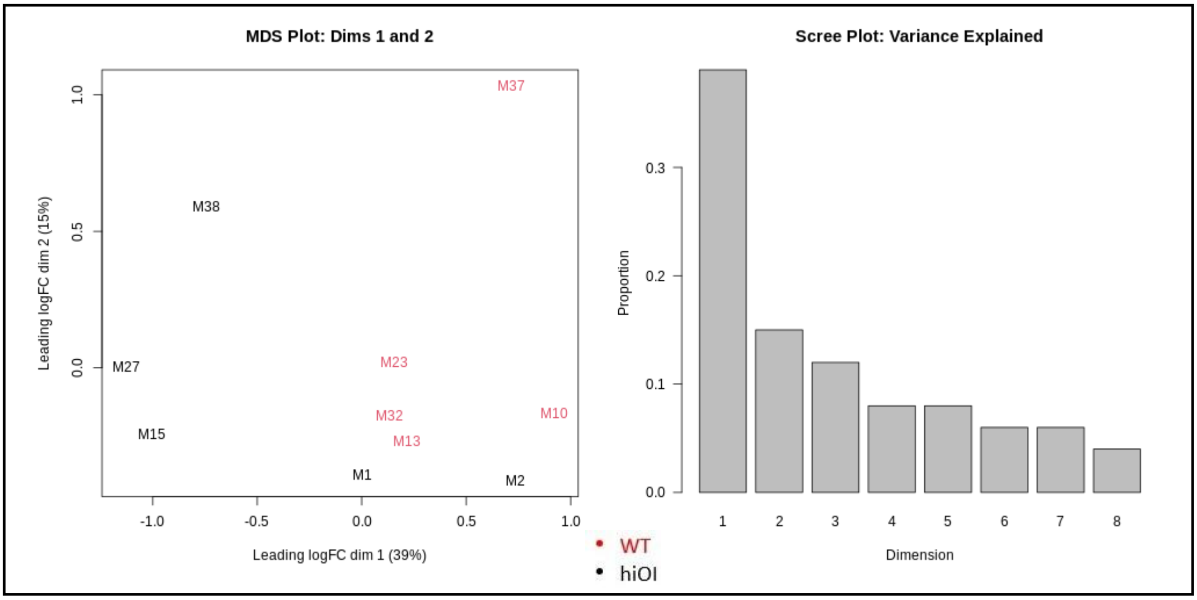
MDS Plot: Dims 1 and 2
Leading logFC dim 2 (15%)
Leading logFC dim 1 (39%)
M37
M38
M27
M23
M32
M10
M15
M13
M1
M2
-1.0
-0.5
0.0
0.5
1.0
WT
hiOI
Scree Plot: Variance Explained
Proportion
Dimension
0.0
0.1
0.2
0.3
1
2
3
4
5
6
7
8

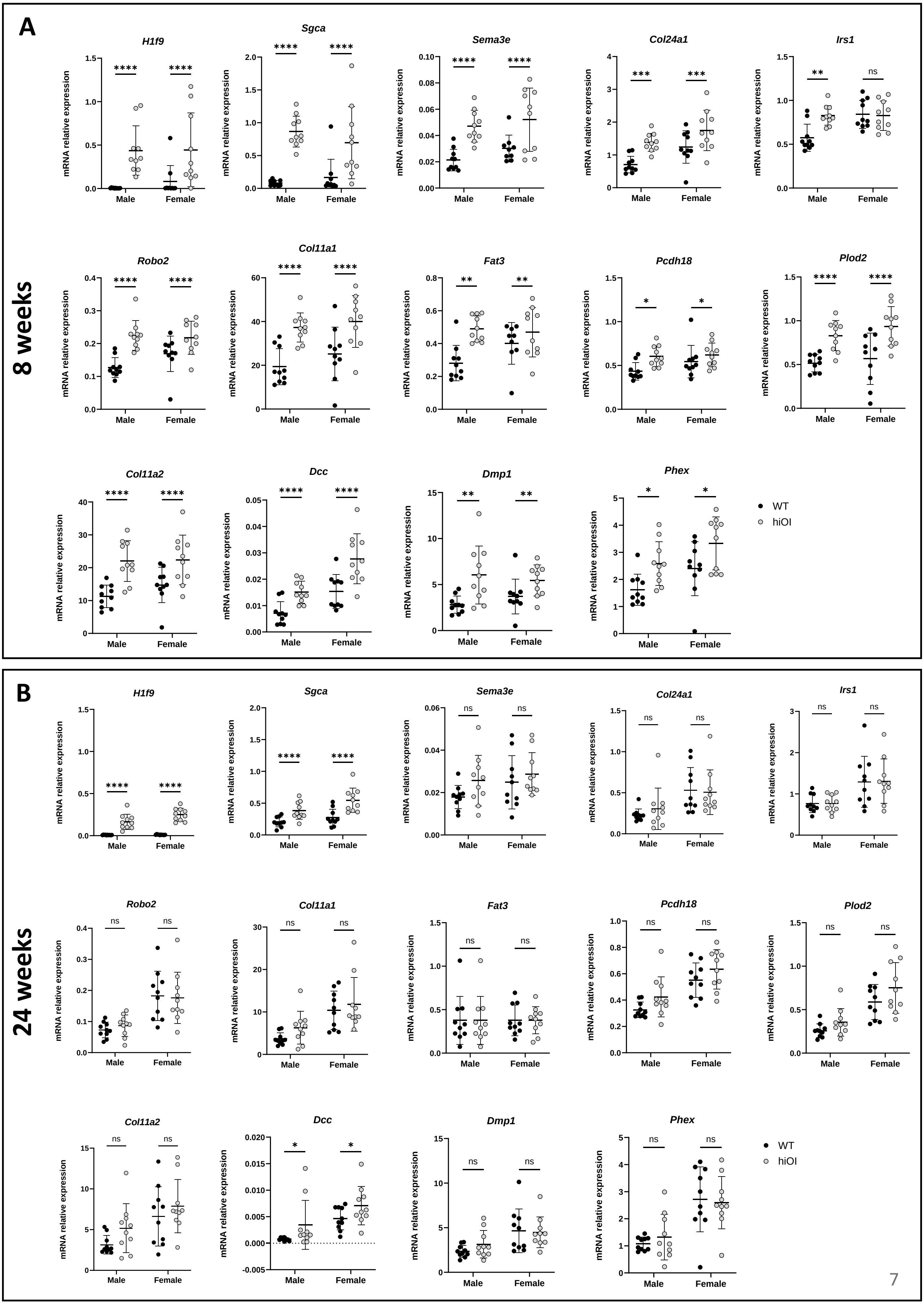
A
8 weeks
H1f9
Sgca
Sema3e
Col24a1
Irs1
Robo2
Col11a1
Fat3
Pcdh18
Plod2
Col11a2
Dcc
Dmp1
Phex
mRNA relative expression
Male
Female
WT
hiOI
B
24 weeks
H1f9
Sgca
Sema3e
Col24a1
Irs1
Robo2
Col11a1
Fat3
Pcdh18
Plod2
Col11a2
Dcc
Dmp1
Phex
mRNA relative expression
Male
Female
WT
hiOI

Fig. S4

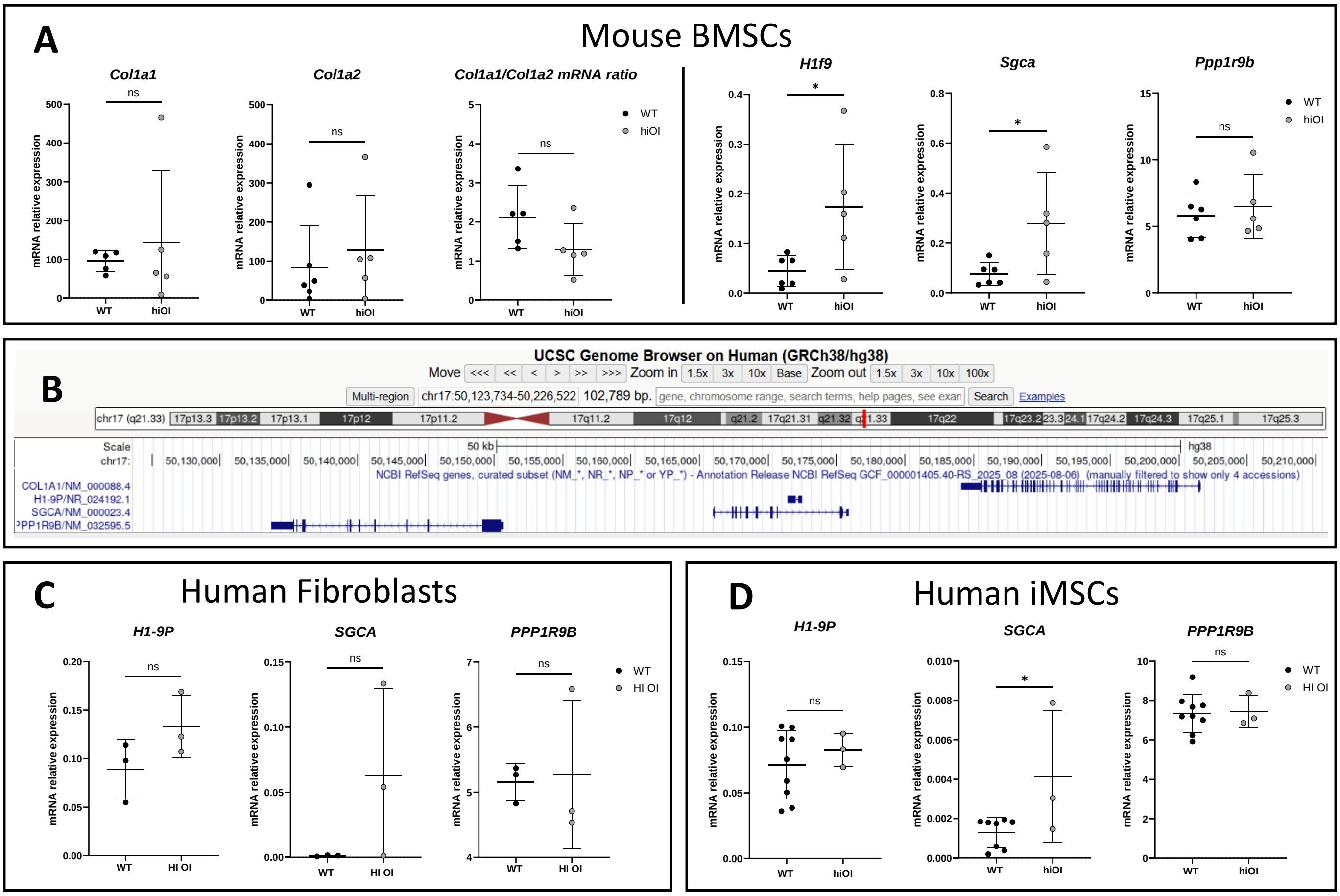
A
Mouse BMSCs
Col1a1
Col1a2
Col1a1/Col1a2 mRNA ratio
H1f9
Sgca
Ppp1r9b
mRNA relative expression
WT
hiOI
ns
*
B
UCSC Genome Browser on Human (GRCh38/hg38)
chr17:50,123,734-50,226,522 102,789 bp.
NCBI RefSeq genes, curated subset (NM_*, NR_*, NP_* or YP_*) - Annotation Release NCBI RefSeq GCF_000001405.40-RS_2025_08 (2025-08-06) (manually filtered to show only 4 accessions)
COL1A1/NM_000088.4
H1-9P/NR_024192.1
SGCA/NM_000023.4
PPP1R9B/NM_032595.5
C
Human Fibroblasts
H1-9P
SGCA
PPP1R9B
HI OI
D
Human iMSCs

Fig. S5

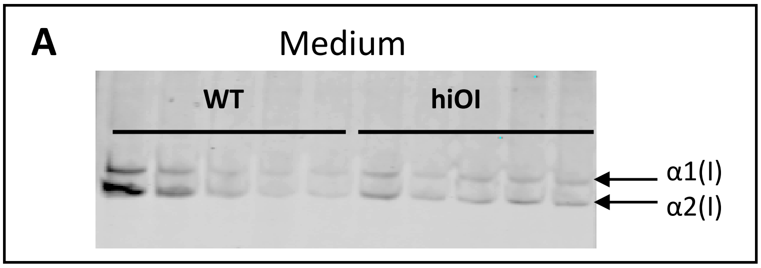

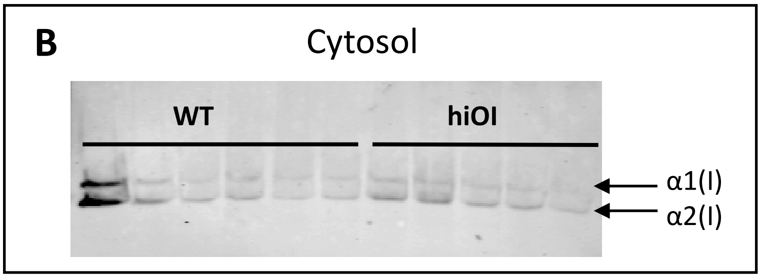

C

	% Reduction in hiOI	
**Protein/Fraction**	**Medium**	**Cytosol**
α1(I)	25.4%	24.9%
a2(I)	46.0%	24.7%
Total Collagen type I	39.9%	23.0%
Ratio α1(I)/a2(I)	1.3%	19.4%

Fig. S6

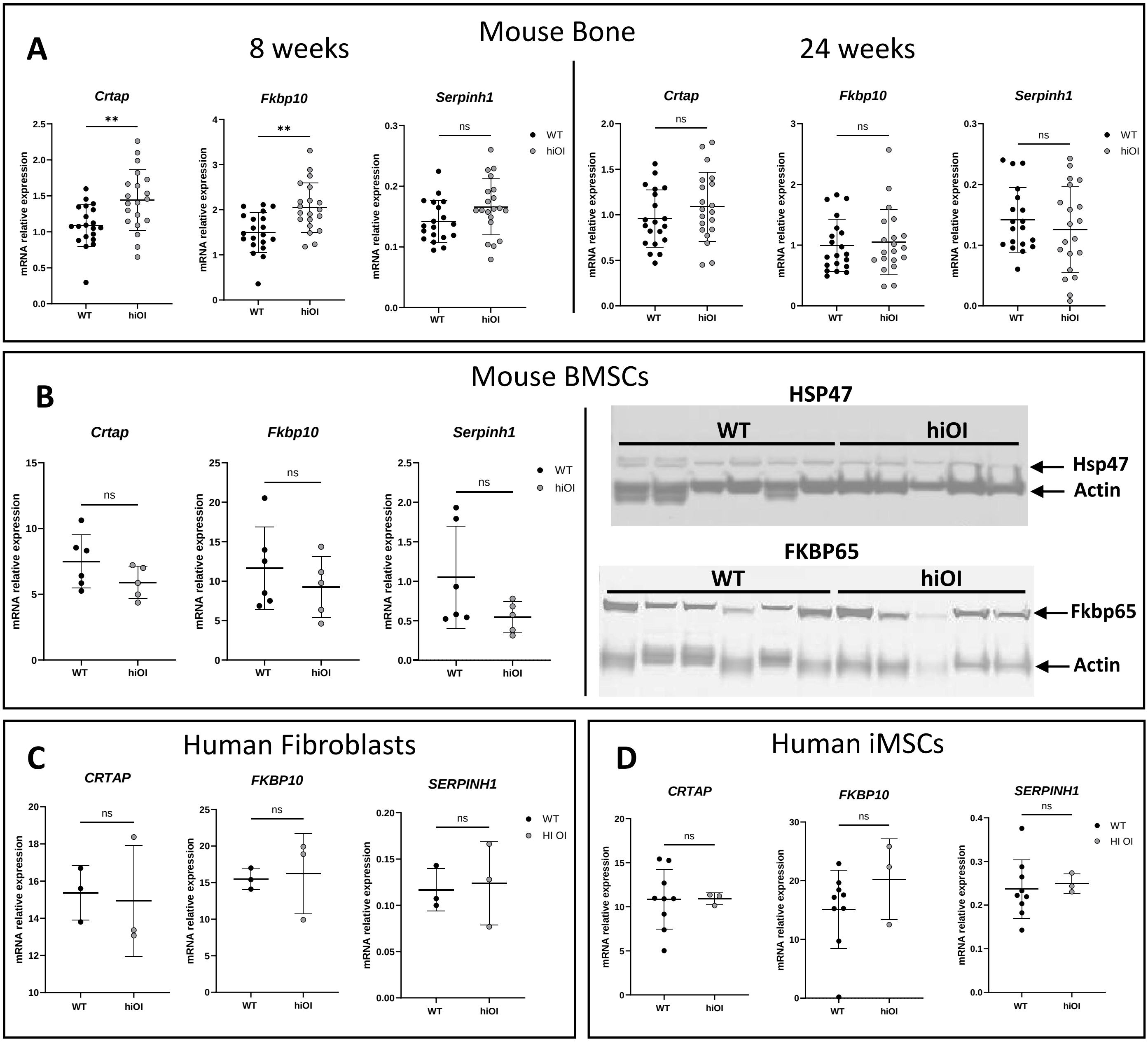
A
Mouse Bone
8 weeks
24 weeks
Crtap
Fkbp10
Serpinh1
mRNA relative expression
WT
hiOI
**
ns
B
Mouse BMSCs
Crtap
Fkbp10
Serpinh1
HSP47
WT
hiOI
Hsp47
Actin
FKBP65
Fkbp65
C
Human Fibroblasts
CRTAP
FKBP10
SERPINH1
HI OI
D
Human iMSCs
CRTAP
FKBP10
SERPINH1

Supplementary Figure Legends

Fig. S1. Bone properties of hiOI mice at the ages of 8 and 24 weeks. (A) Micro-computed tomography (micro-CT) showed reduced trabecular bone quantity in 8-week-old animals. **(B)** Micro-CT of femora from 24-week-old animals showed partial improvement in bone quality and mass in hiOI mice. **(C)** Histomorphometry of Goldner-stained sections from male 8-week-old mice shows a decrease in both cortical and trabecular indices. **(D)** The mechanoproperties of hiOI bones are also impaired, as demonstrated by the 3-point bending test. All comparisons of WT to hiOI data were measured with unpaired t-test. Gender subgroups were analyzed using a two-way ANOVA test with genotype as the main effect and multiple comparisons. P-values are specified as follows: p≤0.05 (*), p≤0.01 (**), p≤0.001 (**), p≤0.0001 (****).

Fig. S2. MDS and Scree plots of bone RNA sequencing in hiOI and WT mice.

Fig. S3. Validation of differentially expressed genes identified by RNA sequencing using RT-qPCR. Real-Time Quantitative PCR (RT-qPCR) analysis was used to validate the top 14 upregulated genes identified by RNA Seq in 20 hiOI and 20 WT mice at **(A)** 8 weeks and **(B)** 24 weeks of age. Gender subgroups were analysed using a two-way ANOVA with multiple comparisons. P-values are indicated as the following: P-values ≤0.05 (*), ≤ 0.01 (**), ≤ 0.001 (***), ≤0.0001 (****).

Fig. S4. Expression analysis of *COL1A1* locus-associated genes in mouse bone marrow stromal cells (BMSCs), human fibroblasts, and human induced mesenchymal stem cells (iMSCs). (A) RT-qPCR analysis of *Col1a1*, *Col1a2*, their expression ratio, and genes located adjacent to the *Col1a1* locus (*H1f9*, *Sgca*, *Ppp1r9b*) in BMSCs derived from WT and hiOI mice at 8 weeks of age. **(B)** Schematic of the human *COL1A1* locus on chromosome 17, showing adjacent genes *H1-9p*, *SGCA* and *PPP1R9B.* Screenshot captured from the UCSC Genome Browser (https://genome.ucsc.edu/). **(C-D)** Real-time quantitative PCR analysis of *H1-9p*, *SGCA* and *PPP1R9B* expression in human fibroblasts **(C)** and iMSCs **(D)**. All comparisons of WT to hiOI data were measured with unpaired t-test. P-values ≤0.05 (*).

Fig. S5. Western blot analysis of collagen type I chains in WT and hiOI mouse BMSCs. (A-B) Western blots showing the α1(I) and α2(I) protein bands of collagen extracted from BMSC-conditioned medium **(A)** and cytosolic fractions **(B)**. **(C)** Quantification of α1(I), α2(I), total collagen type I, and the α1(I)/α2(I) protein ratio, presented as the average percentage reduction in hiOI relative to WT.

Fig. S6. Expression analysis of collagen post-translational processing genes in mouse bone, mouse BMSCs, human fibroblasts and human iMSCs. (A) RT-qPCR analysis of *Crtap*, *Fkbp10* and *Serpinh1* in mouse bone tissue at 8 and 24 weeks of age. **(B)** RT-qPCR analysis of *Crtap*, *Fkbp10* and *Serpinh1* in mouse BMSCs, along with Western blot analysis of Hsp47 and Fkbp65 proteins. Actin was used as a loading control. **(C–D)** RT-qPCR analysis of *CRTAP*, *FKBP10* and *SERPINH1* in human fibroblasts (**C**) and human iMSCs (**D**). All comparisons between WT and hiOI samples were assessed using unpaired *t*-tests. *P*≤0.01 (**).

Supplementary Tables

Table S1. Details of age, sex, and number of mice used for each experiment.

Experiment	Mouse age	Male animals	Female animals
Body weight measurement	8 weeks	10 hiOI, 10 WT	10 hiOI, 10 WT
	24 weeks	10 hiOI, 10 WT	10 hiOI, 10 WT
Femur length measurement	8 weeks	10 hiOI, 10 WT	10 hiOI, 10 WT
	24 weeks	10 hiOI, 10 WT	10 hiOI, 10 WT
Micro-CT analysis	8 weeks	10 hiOI, 10 WT	10 hiOI, 10 WT
	24 weeks	10 hiOI, 10 WT	10 hiOI, 10 WT
Histomorphometry	8 weeks	10 hiOI, 10 WT	-
CT scan	24 weeks	1 hiOI, 1 WT	1 hiOI, 1 WT
	8 weeks	1 hiOI, 1 WT	1 hiOI, 1 WT
Three-point bending test	8 weeks	9 hiOI, 9 WT	4 hiOI, 8 WT
RNAseq	8 weeks	5 hiOI, 5 WT	-
RT-qPCR	8 weeks	10 hiOI, 10 WT	10 hiOI, 10 WT
	24 weeks	10 hiOI, 10 WT	10 hiOI, 10 WT
P1NP ELISA	8 weeks	10 hiOI, 10 WT	10 hiOI, 10 WT
	24 weeks	10 hiOI, 10 WT	10 hiOI, 10 WT
TRAcP 5b ELISA	8 weeks	10 hiOI, 10 WT	10 hiOI, 10 WT
Western Blot	42 weeks	2 hiOI, 1 WT	1 hiOI, 2 WT

hiOI – Haploinsufficient Osteogenesis Imperfecta; WT – Wild Type.

Table S2. qPCR program.

Step	Tm (°C)	Time (s)	Ramp Rate (°C/s)	Cycles
**Preheat**	95	10 min	4.8	1 ×
**PCR cycling**	95	10	4.8	45 ×
	60	5	2.5	
	72	10	4.8	
	78	5	4.8	
**Melting Curve**	95	1	4.8	1 ×
	60	1	2.2	
	95	1	0.03	
**Cooling**	40	30	1.5	1 ×

Min – Minutes; s – Seconds; Tm – Melting temperature.

Table S3. Mouse and human primer sequences used for RT-qPCR analysis.

Gene	Forward Primer	Reverse Primer	Product Size (bp)
**Mouse**			
BC055402	AGTGTCCATGACCACACGAC	GCCAATCATGAACCGCACTC	116
Col1a1	CGATGGATTCCCGTTCGAGT	TTCGATGACTGTCTTGCCCC	292
Col1a2	AATGGTGGCAGCCAGTTTGA	TCCAGGTACGCAATGCTGTT	143
Col11a1	ACAGTAGCACAAACAGAGGCAA	AATCCCTGCCGTCTACTCCT	229
Col11a2	AAGAGCAAGCAGCACTGACA	CCTCTTCAGTGGGTCGATGT	273
Col24a1	ATGCATTTAGGAGCCTACAG	GATGGCGTCATCGGGGTAAT	292
Crtap	AACCAAGTCGTACGAGAGCC	TGGTACATGGTCGCCACAAA	291
Dcc	AAGGTCAAGCACGTGGAGCA	ACGAAGCACATCCACAGAGG	82
Dmp1	CTTGTGTTCCTTTGGGGGCT	GACTGTCAGATCCTTGGGAGCA	232
Fat3	ATGCAATGTGTGGGCTATGA	AACGACAATAGCAGCCGTGA	171
Fkbp10	TCGACTTCCACAACCCTTCG	CCTCATAGTCGTGGGACGAG	159
H1f9	ATACGCAGCCTCAAATGGCT	CACATACGACATGGTGGGCT	137
Irs1	TGGTTCAGTCTCTTCCTCATGCC	TCACGAGAGGTGACTCAATA	125
Pcdh18	AAGGCCATCCAGGCAGATTC	GCGCAGAGCTAGCTTGAAGT	204
Phex	TTGGCAACGTACTGCAAACC	GTCTGTACTGGCCTCTGTCG	307
Plod2	CAAATCATAAAATCGTCTTTGCAG	TTCTCTGGCACGTGGTTTGA	221
Ppp1r9b	AGAAGCTGGGCATCTTCGTC	CCTGACTGGTTGGCAGTTGT	179
Robo2	TTTTACTCCAACCCCTGCAC	CGTCCAGAACCTCTCAGTCTCC	212
Sema3e	TGTGCGTGAATGACATGGGA	ATGACACCAGGCAAAGGGAG	401
Sgca	CGAGTAGACAGCGCACAGAT	CACATACGACATGGTGGGCT	107
Slc9a2	CGGGTCATTGAGCCACTCTT	GCATCAGTGTTTCCTGGGAT	483
Serpinh1	GAGCAGAGCCTGTCTGAGGA	GCCTTTTTCATTCTGGGCCG	74
Tbp	CCTATCACTCCTGCCACACC	ATGACTGCAGCAAATCGCTTG	161
**Human**			
COL1A1	GTGCTAAAGGTGCCAATGGT	ACCAGGTTCACCGCTGTTAC	128
COL1A2	GTGGCAGTGATGGAAGTGTG	GATTACCAGGAGGTCCAACG	198
CRTAP	TGAGCACTTCCAGCCCAGAC	TCCACAACTTCTCCCTCATCATCA	120
FKBP10	GACCTGCAATGAGACCACCA	CCGTAGTCATGCGAGGTGAA	99
H1-9P	CCACACAGGGAGAGAAGCAG	CAGCCCTCAGGATCACCTTG	182
PPP1R9B	AGAAGCGTGTGGAGAGGTTG	CGGCCAATCATAAACCGCAC	289
SGCA	TTGCTACGACACCTTGGCAC	CCAGGTCTCTCTTCAGCCTTC	281
SERPINH1	AGCGTTTCCAACTTTCCAGA	CCTGCCTTTTTCATTCTGGGTC	146
TBP	AGTTCTGGGATTGTACCGCA	TCCTCATGATTACCGCAGCA	139

Table S4. mRNA expression of *Col1a1, Col1a2* and *Col1a1*/*Col1a2* mRNA expression ratio in individual animals at 8 and 24 weeks of age.

**Sample**	**Age (weeks)**	**Gender**	**Genotype**	***Col1a1***	***Col1a2***	***Col1a1/Col1a2***
M1	8	M	hiOI	647.5	583.5	1.110
M2	8	M	hiOI	642.0	546.4	1.175
M3	8	F	WT	NA	NA	NA
M4	8	M	WT	470.6	249.3	1.888
M5	8	F	hiOI	709.5	626.1	1.133
M6	8	F	hiOI	824.9	733.4	1.125
M7	8	F	hiOI	746.1	631.0	1.182
M8	8	F	WT	895.1	412.6	2.169
M9	8	M	WT	495.1	247.7	1.999
M10	8	M	WT	486.8	229.1	2.125
M11	8	F	hiOI	577.0	525.7	1.098
M12	8	F	hiOI	332.0	279.4	1.188
M13	8	M	WT	746.1	313.3	2.381
M14	8	M	hiOI	1126	751.2	1.499
M15	8	M	hiOI	901.4	659.4	1.367
M16	8	F	WT	699.3	312.9	2.235
M17	8	F	WT	895.5	380.3	2.355
M18	8	F	WT	679.4	284.6	2.387
M19	8	M	WT	688.0	280.1	2.456
M20	8	F	WT	1191	466.5	2.553
M21	8	F	hiOI	766.6	569.2	1.347
M22	8	M	hiOI	779.5	498.0	1.565
M23	8	M	WT	643.7	267.0	2.411
M24	8	M	WT	1108	397.0	2.791
M25	8	M	hiOI	664.7	578.1	1.150
M26	8	M	WT	901.7	398.3	2.264
M27	8	M	hiOI	512.6	429.6	1.193
M28	8	M	hiOI	724.4	609.4	1.189
M29	8	F	hiOI	1184	491.2	2.410
M30	8	F	WT	766.6	532.1	1.441
M31	8	M	hiOI	822.8	577.5	1.425
M32	8	M	WT	743.7	327.1	2.274
M33	8	F	WT	677.2	278.8	2.429
M34	8	F	hiOI	762.7	665.3	1.146
M35	8	F	hiOI	465.4	390.0	1.193
M36	8	F	WT	983.9	429.7	2.290
M37	8	M	WT	546.4	160.4	3.406
M38	8	M	hiOI	555.5	476.6	1.166
M39	8	F	WT	1027	412.9	2.487
M40	8	F	hiOI	895.7	665.3	1.346
M1	24	M	WT	128.5	75.88	1.693

M2	24	M	hiOI	120.2	95.54	1.258
M3	24	M	WT	80.97	86.14	0.940
M4	24	M	hiOI	88.85	78.87	1.127
M5	24	F	WT	760.2	252.0	3.017
M6	24	F	WT	232.7	95.95	2.425
M7	24	M	WT	280.8	121.1	2.319
M8	24	M	WT	136.9	69.11	1.981
M9	24	F	WT	437.0	169.1	2.584
M10	24	F	WT	269.8	114.9	2.348
M11	24	F	WT	265.1	123.7	2.143
M12	24	F	hiOI	205.2	159.5	1.287
M13	24	F	hiOI	283.2	196.9	1.438
M14	24	F	WT	226.8	80.08	2.832
M15	24	F	hiOI	1370	100.8	1.359
M16	24	F	hiOI	195.5	145.5	1.344
M17	24	M	hiOI	36.12	32.72	1.104
M18	24	M	WT	159.8	61.93	2.580
M19	24	M	hiOI	60.33	50.12	1.204
M20	24	F	hiOI	245.5	205.2	1.196
M21	24	F	hiOI	103.7	104.4	0.993
M22	24	F	hiOI	106.7	83.18	1.283
M23	24	M	WT	250.4	102.8	2.436
M24	24	M	hiOI	136.7	108.2	1.263
M25	24	M	WT	115.3	41.86	2.754
M26	24	M	WT	102.8	42.62	2.412
M27	24	F	hiOI	137.4	93.88	1.464
M28	24	F	WT	176.0	75.58	2.329
M29	24	F	WT	381.2	165.9	2.298
M30	24	M	hiOI	124.1	102.0	1.217
M31	24	M	hiOI	278.2	218.7	1.272
M32	24	F	hiOI	371.2	277.8	1.336
M33	24	M	hiOI	125.2	128.7	0.973
M34	24	M	hiOI	170.6	115.8	1.473
M35	24	M	hiOI	203.8	148.4	1.373
M36	24	F	hiOI	412.8	271.9	1.518
M37	24	M	WT	233.0	66.10	3.525
M38	24	F	WT	505.9	195.3	2.590
M39	24	F	WT	517.3	189.9	2.724
M40	24	M	WT	111.3	55.69	1.999

F – Female; hiOI – Haploinsufficient Osteogenesis Imperfecta; M – Male; NA – Not Available; w – weeks; WT – Wild Type.
